# Novel 9-Benzylaminoacridine Derivatives as Dual Inhibitors of Phosphodiesterase 5 and Topoisomerase II for the Treatment of Colon Cancer

**DOI:** 10.3390/molecules28020840

**Published:** 2023-01-14

**Authors:** Lina Ammar, Hung-Yu Lin, Shou-Ping Shih, Tsen-Ni Tsai, Yu-Ting Syu, Mohammad Abdel-Halim, Tsong-Long Hwang, Ashraf H. Abadi

**Affiliations:** 1 Department of Pharmaceutical Chemistry, Faculty of Pharmacy and Biotechnology, German University in Cairo, Cairo 11835, Egypt; 2 School of Medicine, College of Medicine, I-SHOU University, Kaohsiung 824, Taiwan; 3 Division of Urology, Department of Surgery, E-Da Cancer & E-Da Hospital, I-SHOU University, Kaohsiung 824, Taiwan; 4 Doctoral Degree Program in Marine Biotechnology, National Sun Yat-Sen University (NSYSU), 70 Lien-Hai Road, Kaohsiung 80424, Taiwan; 5 Doctoral Degree Program in Marine Biotechnology, Academia Sinica, 128 Academia Road, Section 2, Nankang, Taipei 11529, Taiwan; 6 Graduate Institute of Marine Biology, National Dong Hwa University, Pingtung 944, Taiwan; 7 Graduate Institute of Natural Products, College of Medicine, Chang Gung University, Taoyuan 333, Taiwan; 8 Research Center for Chinese Herbal Medicine, Graduate Institute of Health Industry Technology, College of Human Ecology, Chang Gung University of Science and Technology, Taoyuan 333, Taiwan; 9 Department of Anesthesiology, Chang Gung Memorial Hospital, Taoyuan 333, Taiwan; 10 Department of Chemical Engineering, Ming Chi University of Technology, New Taipei City 243, Taiwan

**Keywords:** acridine derivatives, anticancer agents, phosphodiesterase 5, topoisomerase II, colorectal carcinoma

## Abstract

It has been shown that phosphodiesterase 5 (PDE5) inhibitors have anticancer effects in a variety of malignancies in both in vivo and in vitro experiments. The role of cGMP elevation in colorectal carcinoma (CRC) has been extensively studied. Additionally, DNA topoisomerase II (Topo II) inhibition is a well-established mechanism of action that mediates the effects of several approved anticancer drugs such as doxorubicin and mitoxantrone. Herein, we present 9-benzylaminoacridine derivatives as dual inhibitors of the PDE5 and Topo II enzymes. We synthesized 31 derivatives and evaluated them against PDE5, whereby 22 compounds showed micromolar or sub-micromolar inhibition. The anticancer activity of the compounds was evaluated with the NCI 60-cell line testing. Moreover, the effects of the compounds on HCT-116 colorectal carcinoma (CRC) were extensively studied, and potent compounds against HCT-116 cells were studied for their effects on Topo II, cell cycle progression, and apoptosis. In addition to exhibiting significant growth inhibition against HCT116 cells, compounds **11**, **12**, and **28** also exhibited the most superior Topo II inhibitory activity and low micromolar PDE5 inhibition and affected cell cycle progression. Knowing that compounds that combat cancer through multiple mechanisms are among the best candidates for effective therapy, we believe that the current class of compounds merits further optimization and investigation to unleash their full therapeutic potential.

## 1. Introduction

Phosphodiesterase 5 (PDE5) inhibitors are a well-established pharmacological class that act by increasing intracellular levels of the second messenger cGMP [1]. Due to their effect on smooth muscles, PDE5 inhibitors have been approved for the treatment of erectile dysfunction and pulmonary hypertension [2,3,4]. There are several PDE5 cGMP-competitive inhibitors that have been marketed, such as sildenafil, tadalafil, vardenafil, and avanafil [5,6,7,8]. Recently, new classes with non-classical binding properties to PDE5 have been described [9,10,11]. There is emerging evidence from in vivo and in vitro experiments that suggests that PDE5 inhibitors have anticancer effects in many types of cancers, such as chronic lymphocytic leukemia, prostate, colorectal, brain, breast, and lung cancers [11,12,13,14]. Interestingly, PDE5 expression and activity have been shown to be important for colon tumor cell growth and survival [15,16]. Moreover, the use of sildenafil in a series of human colorectal cancer cell lines (HT-29, SW-480, SW-620, HCT-116, and SW-1116) was shown to affect the proliferation and apoptosis of cancer cells in in vitro experiments [17]. Tumor volume was also significantly reduced in human cancer cell SW-480 and HCT-116 xenograft experiments [18]. Additionally, PDE5 inhibitors showed strong anticancer effects and reduced metastasis in colorectal cancer patients after reoperation [19]. Previous studies have suggested that inhibition of PDE5, and the consequent increase in cellular cGMP, activates PKG, which in turn mediates the phosphorylation and subsequent proteolysis of β-catenin (an oncogenic protein which is responsible for the transcription of important growth regulatory genes that enhance tumor cell proliferation and survival) [20,21]. Furthermore, sildenafil and vardenafil were shown to reduce the resistance of tumor cells to chemotherapeutic agents through their action on efflux transporters [22,23], indicating a synergetic effect in combining PDE5 inhibitors with anticancer agents for treating cancer [24].

DNA-damaging agents have long been used in cancer chemotherapy and are commonly used to treat a variety of cancers, including hematological malignancies, various solid tumors (lung, prostate, breast, bladder, colorectal, etc.), and soft tissue sarcomas [25,26]. Many of these agents target the DNA topoisomerase enzymes, which are mainly classified into topoisomerase I (Topo I) and topoisomerase II (Topo II). Both Topo I and Topo II catalyze DNA break and reseal to enable the replication and transcription of DNA, which is crucial for cell proliferation [26,27]. Topo II was found to be more important as a drug target [28,29,30]. The current clinical drugs targeting Topo II can be divided into Topo II poisons and Topo II catalytic inhibitors according to the nature of their action. Drugs clinically used as topo II poisons are etoposide, doxorubicin, daunorubicin, and mitoxantrone, which act on the covalent complex between Topo II and DNA by blocking DNA replication and transcription, causing increased levels of DNA damage in cells and promoting apoptosis [31,32,33]. In contrast, the action of topo II catalytic inhibitors, such as dexrazoxane and merbarone, is mediated by inhibiting the catalytic function of Topo II without causing DNA damage [34].

**Figure 1:**
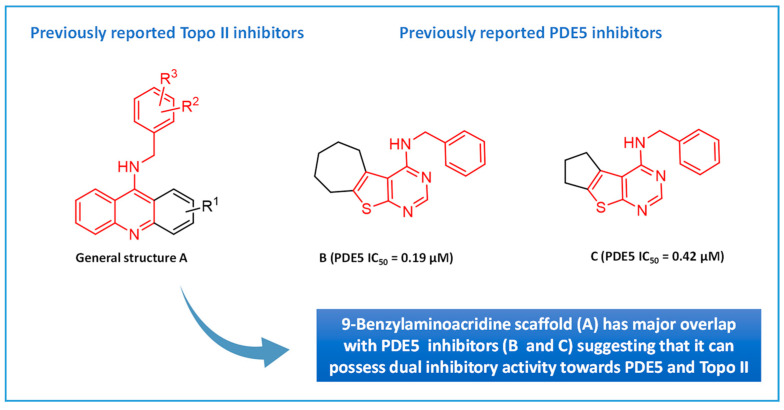
General structure representing 9-benzylaminoacridines as well as our previously reported thienopyrimidine PDE5 inhibitors.

Given that the etiology of cancer is multifaceted, the use of a molecule that can hit several targets might offer a better therapeutic potential than the classical ‘’one target-one molecule’’ approach [35]. To fulfill this purpose, a class of anticancer compounds that act by hitting more than one target for cancer chemotherapy was designed. Several 9-benzylaminoacridine derivatives have been reported as inhibitors of Topo II, as presented by general structure A in Figure 1 [34,36]. It was noticed that their general scaffold possesses common features (colored in red) that coincide with the scaffold of our previously reported thienopyrimidine PDE5 inhibitors, compounds **B** and **C**, as shown in Figure 1 [37]. This finding suggested that new compounds that have the general scaffold **A** can be synthesized aiming to combat cancer via the dual inhibition of Topo II and PDE5.

## 2. Results and Discussion

### 2.1. Chemistry

**Scheme 1:**
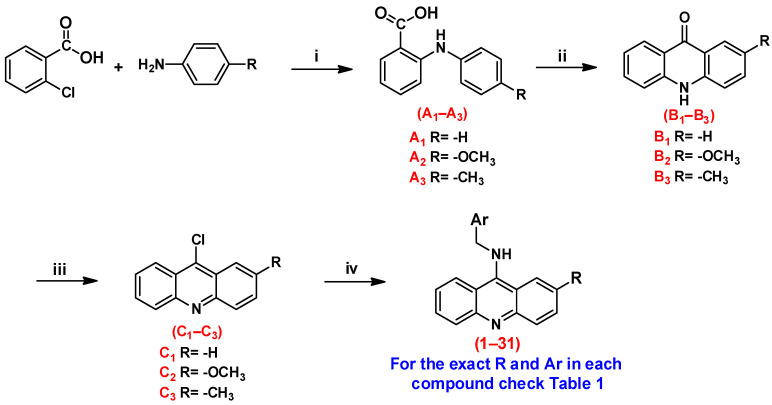
Reagents and conditions. (i) Cu, K_2_CO_3,_ KI, and DMF, reflux overnight. (ii) H_2_SO_4_, 100–110 °C for 4 h. (iii) POCl_3_, 100–110 °C for 3 h. (iv) Benzyl amine derivatives K_2_CO_3_, KI, and ethanol, reflux overnight.

The syntheses of the planned compounds were achieved through a four-step synthesis as illustrated in Scheme 1. The synthesis of substituted *N*-phenylanthranilic acids (**A1**–**A3**) was carried out using the Ullmann reaction between 2-chlorobenzoic acid and the respective aniline derivative in the presence of copper as a catalyst under high-temperature conditions. Subsequently, the *N*-phenylanthranilic acid was self-condensed with conc. sulfuric acid to produce acridones (**B1**–**B3**), which were then converted to the respective 9-chloroacridines (**C1**–**C3**) via reflux in POCl_3_. The target compounds were obtained by reacting benzyl amine analogs with 9-chloroacridine derivatives in ethanol under reflux in the presence of potassium carbonate as a base.

### 2.2. Biological Evaluation 

#### 2.2.1. Activity against PDE5

All the synthesized acridine derivatives were tested for their in vitro ability to inhibit PDE5A at a screening dose of 10 µM. IC_50_ values were determined for compounds exhibiting percentage inhibitions above 50%. The results are shown in Table 1. 

Structure–activity relationship of the PDE5 inhibitory activity: Compound **1** was initially synthesized with an unsubstituted benzyl giving an IC_50_ = 9.33 μM, indicating that our assumption about the possible PDE5 inhibition by this scaffold was correct. Compounds with different substituents on the benzyl ring were synthesized in attempts to enhance their potency, as discussed below.

Monosubstitution of the phenyl ring: Monosubstitution with electron-donating groups was investigated, starting with the lipophilic, electron-donating methyl group in the *ortho*, *meta*, and *para* positions, as seen in compounds **2**, **3**, and **4**, respectively. The methyl group in the *para* position showed slightly higher activity (compound **4**, IC_50_ = 4.44 μM) compared with the *ortho* (**2**) and *meta* (**3**) isomers, which exhibited IC_50_ values of 9.87 μM and 6.40 μM, respectively. On the other hand, the use of the more polar electron-donating methoxy group resulted in the deterioration of the inhibitory activity whether in the *ortho*, *meta,* or *para* positions, as exhibited by compounds **5**, **6**, and **7**, respectively.

Mono-halogen substitutions with fluorine and chlorine were made at different positions of the phenyl ring to produce compounds **8**–**13**. Fluoro- and chloro-substituents in the *para* position (compounds **10** and **13**, respectively) reduced the inhibitory activity compared with the unsubstituted benzyl in compound **1**. Meanwhile, similar inhibitory activity was exhibited by compounds that had either of these substituents in the *meta* position (**9** and **12**, IC_50_ = 6–7 µM). However, the fluoro-substituent in the *ortho* position (compound **8**, IC_50_ = 7.16 μM) showed activity 1.5 times lower than that of the chloro-substituent in the same position (compound **11**, IC_50 =_ 4.33 μM).

Disubstitution of the phenyl ring: All compounds containing dimethoxy phenyl (**16**–**18**), similar to the mono-substituted methoxy analogs, did not show any improvement in the activity.

Additionally, disubstitution of the phenyl ring with fluorine at variable positions (compounds **20, 21**, and **22**) led to a reduction in potency. Exceptionally, compound **20** (2,3-difluorobenzyl) showed an IC_50_ of 7.25 μM, which was comparable to the mono-substituted *ortho* and *meta* fluoro-analogs. While the methoxy group failed to boost the activity against PDE5 in mono- and disubstituted methoxy compounds, combining the *p*-methoxy with *m*-fluoro in compound **19** (3-fluoro-4-methoxy) seemed to have a synergetic effect that resulted in an improvement in the activity (IC_50_ = 3.39 μM). 

Replacement of the phenyl ring from the benzylamino group: Modifications in the structure via the replacement of the phenyl ring with furan, thiophene, and 4-(2-thiophenephenyl) gave rise to compounds **14**, **15**, and **24**, respectively. This resulted in some improvement in the PDE5 inhibitory activity compared with compound **1**, with the exception of compound **24**, which exhibited reduced activity.

Increasing the spacer length: Compound **23** demonstrated the effect of increasing the linker length in the benzylamino moiety via its replacement with phenylethyl amino. This modification showed some improvement in the activity (IC_50_ = 6.68 μM) compared with the benzyl derivative, compound **1**, exhibiting an IC_50_ = 9.33 μM.

Substitution at C2 of the acridine: A few derivatives were synthesized to have methoxy or methyl substituent at C2 of the acridine. These modifications were adopted together with the use of different *para*-monosubstituted benzylamino groups at C9. The methyl group in the *para* position of the benzylamino along with a methoxy group at C2 of the acridine exhibited the highest activity against PDE5 in the present series (compound **26**, IC_50_ = 0.83 μM), indicating that the methoxy group at C2 can be a key modification for boosting the inhibitory potency of the scaffold against PDE5. This observation was further confirmed by comparing compound **25** (containing 2-methoxy acridine and an unsubstituted benzylamino, IC_50_ = 3.61 μM) with compound **1** (IC_50_= 9.33 μM), the respective analog with unsubstituted acridine. Such a comparison showed that the methoxy at position two led to about a three-fold improvement in the activity. Ultimately, the scaffold exhibited low micromolar inhibition against PDE5 with several substituents tolerated on the benzyl moiety. Moreover, the use of a methoxy substituent at position two of the acridine boosted the inhibitory activity to sub-micromolar values. 

#### 2.2.2. Testing the Anticancer Activity of the Compounds

##### NCI 60-Cell Line Testing

The newly synthesized compounds were screened for their in vitro anticancer activity at the Developmental Therapeutic Program (DTP) of the National Cancer Institute (NCI). The screening involved a panel of 60 human tumor cell lines consisting of 9 tissue types to screen for potential anti-cancer agents. The primary screening of submitted compounds was performed by testing a single dose (10^−5^ M) in the full NCI-60 panel. The values for the mean % growth inhibition of the 60 cell lines by compounds (**1**–**14**) and (**16**–**31**) are shown in Table 2. The detailed % inhibition by the tested compounds against the 60 cell lines is shown in Tables S1–S3 in the Supporting Information.

One dose assay was conducted on 30 compounds accepted by the NCI (**1**–**14** and **16**–**31**). Almost all synthesized compounds exhibited a mean growth inhibition above 50% and exhibited high activity against some of the investigated cell lines. The 2-methoxy acridine along with 4-fluoro- (**28**) or 4-chloro- (**29**) on the benzyl ring showed high mean % growth inhibition (92.16 and 93.27%, respectively). Moreover, the unsubstituted acridine with the extended 4-(2-thienyl) benzylamino (**24**) showed the highest mean growth inhibition at 96.88%. This suggests that the electron-donating group at C2 of the acridine with the electron-withdrawing group in the *para* position of the benzylamino (compounds **28** and **29**) or the unsubstituted acridine with the extended aryl benzylamino (compound **24**) are important for anticancer activity.

##### Effects of Compounds on Proliferation of Human Colorectal Cancer Cells and Non-Cancer Cells

Since HCT-116 CRC cells are among the most studied cells in the context of the anticancer activity of PDE5 inhibitors [38,39], the compounds were screened against this cancer cell line for more detailed investigations. Additionally, human non-malignant cells (CCD-966SK) were used as a control cell line to study the selectivity of the compounds (Table 3). The results showed that 19 compounds had a significant inhibitory effect on cell survival, among which compounds **1**–**4**, **10**, **8**, **12**, **20**, **21**, and **28** exhibited high cytotoxicity (IC_50_ < 5 µM). Compounds **5**, **9**, **14**, **11**, **24**, **25**, and **31** were found to have moderate toxicity to HCT-116 cells (IC_50_ < 10 µM). Compounds **2**, **3**, **10**, and **28**, however, were toxic to human non-malignant dermal fibroblast CCD-966SK cells. Conclusively, the above results indicate that the cytotoxicity of compounds **1**, **3**, **4**, **8**, **12**, **20**, and **21** was more selective for HCT-116 cells. 

The prominent structural features that enhanced the anticancer activity against HCT-116 cells are certainly worth mentioning. It should be noted that having *ortho-* and/or *para*-fluorinated benzyl groups at position nine of the acridine is an important feature in many potent growth inhibitors. This could be observed in compounds **8**, **10**, **21**, and **28**, such that all these compounds showed an IC_50_ < 4 µM. These compounds had 2-fluorobenzyl, 4-fluorobenzyl, 2,4-difluorobenzyl, and 4-fluorobenzyl substituents, respectively, at position nine of the acridine nucleus. On the other hand, having a *meta-* or *para*-methoxy substituent (alone or in combination with other substituents) at the 9-benzyl moiety reduced anticancer activity, as exhibited by compounds **6**, **7**, **16**, **17**, **18**, **19**, and **27** (with ≤50% growth inhibition at 10 µM). 

#### 2.2.3. Effects of Compounds on Topo II Activity

**Figure 2:**
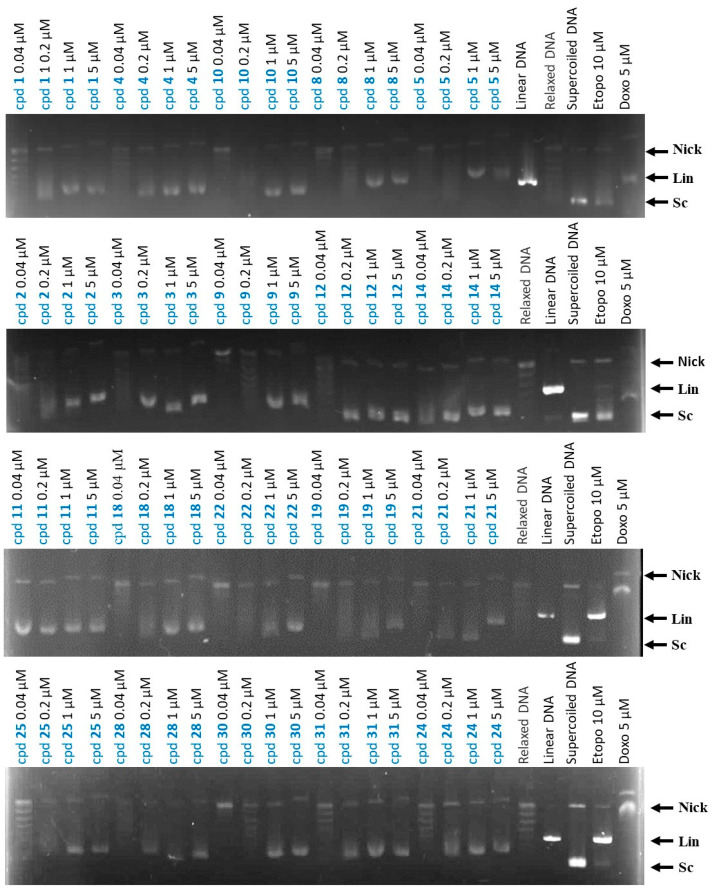
Effects of compounds on topoisomerase II activity. Effects on Topo II-mediated relaxation of supercoiled pHOT1 plasmid DNA in a cell-free system. Positive control: etoposide (10 µM); doxorubicin (5 µM) used as Topo II poison (induction of linear DNA); DNA relaxation: plasmid DNA + topo IIα + solvent control. Nick, nicked DNA; Sc, supercoiled DNA; Lin, linear DNA.

It has been previously reported that 9-benzylaminoacridines inhibit Topo II [34]. Consequently, the effects of the synthesized compounds on Topo II activity were evaluated with a cell-free assay using a purified human DNA Topo II-protein-mediated relaxation of the negatively supercoiled pHOT1 plasmid DNA [40]. Compounds with cytotoxicity against HCT-116 (IC_50_ < 10 µM) were evaluated for their activity against Topo II. Two Topo II inhibitors (doxorubicin and etoposide) were used as positive controls. The results show that compounds **1**–**5**, **8**, **9**, **11**, **14**, **18**, **20**, **21**, **22**, **24**, **25**, **30**, and **31** at 1 and 5 µM doses have a larger base pair band than nicked DNA, and these results are similar to those of doxorubicin. It is, therefore, speculated that these compounds may insert themselves into DNA and interfere with DNA replication (Figure 2). Notably, compound **11** was found to be the most potent Topo II inhibitor, such that it could dramatically increase the amount of supercoiled DNA at a very low concentration (0.04 µM). This finding suggests that compound **11** inhibits Topo II activity with a higher potency than doxorubicin and that it can, additionally, intercalate into DNA, interfering with DNA replication at high doses. Furthermore, compounds **12** and **28** are considered the second and third best Topo II inhibitors, showing their effects at 0.2 and 1 μM, respectively. 

#### 2.2.4. The Effects of Compounds on Apoptosis and Cell Cycle in HCT-116 Cells

**Figure 3:**
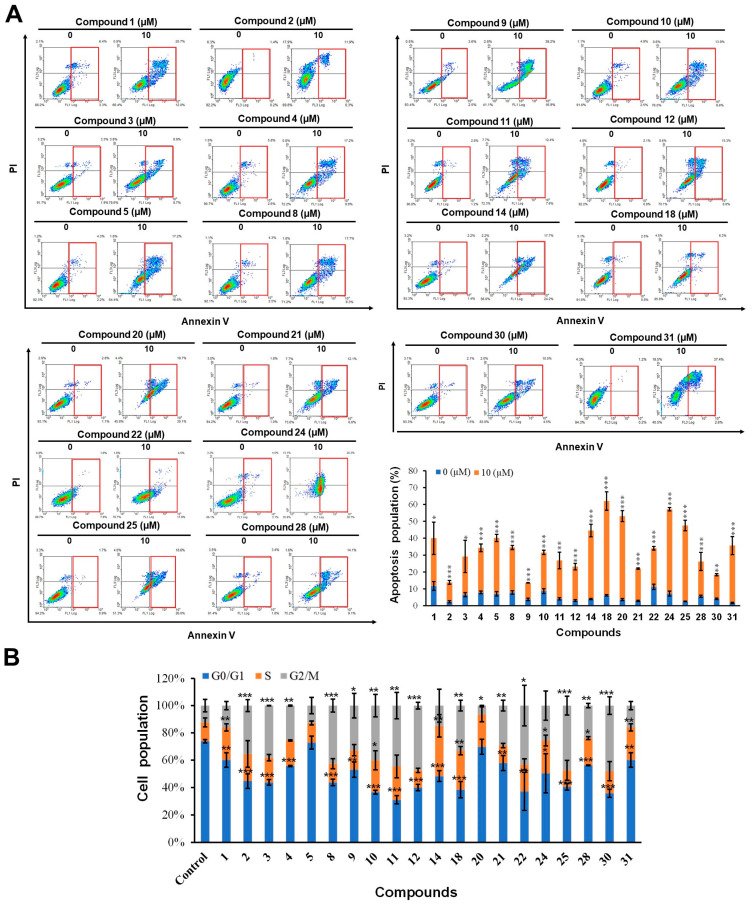
Apoptosis induction and cell cycle effects of compounds in HCT-116 cells. (**A**) The apoptotic population of HCT-116 cells was analyzed using flow cytometry after treatment with 10 µM of the test compound for 24 h followed by Annexin V/propidium iodide (PI) staining. (**B**) After treatment with compound concentrations of 10 µM for 24 h, cells were fixed, stained with propidium iodide (PI), and analyzed for cell cycle distribution. Results are expressed as the means ± SD from three independent experiments (* *p* < 0.05 and ** *p* < 0.01 *** *p* < 0.001, control versus compound).

For the same set of compounds, the apoptosis-inducing effects in HCT-116 cells were assessed using Annexin-V/PI double staining at 10 µM (Figure 3). Compounds **1**–**5**, **8**–**12**, **21**, **22**, **28**, and **30** exhibited only moderate induction of apoptosis (less than 20%), while compounds **14**, **18**, **20**, **24**, **25**, and **31** exhibited significant apoptosis-inducing effects (20%–50%).

A cell cycle analysis showed that most of the compounds affected cell cycle regulation (except for compound **5**). Compounds **1**, **14**, **24**, and **31** arrested the cell cycle in the S phase, while compounds **2**, **3**, **8**, **9**, **11**, **12**, **13**, **22**, **25**, and **30** caused cell cycle arrest in the G2/M phase. In addition, compounds **4**, **10**, **18**, **25**, and **28** increased the number of cells in the S phase and G2/M phase. Finally, compound **20** caused cell cycle arrest in the S phase and reduced the percentage of cells in the G2/M phase. 

To sum up, the above results demonstrate that the compounds have variable effects on cell cycle and apoptosis, such that compounds **14**, **20**, **24**, and **31** can induce apoptosis and arrest the cell cycle in the S phase, while compounds **2**, **8**, **9**, **11**, **12, 21**, **22**, **25**, **30** can cause cell cycle arrest in the G2/M phase and induce apoptosis. Compounds **4**, **10**, **18**, and **28** affect the S phase and G2/M phase of the cell cycle and induce apoptosis. Finally, compound **5** induces apoptosis and does not affect the cell cycle.

## 3. Experimental Section

### 3.1. Chemistry

Solvents and reagents used were obtained from commercial suppliers and were used without further purification. All organic solvents used were of HPLC grade. All starting materials were obtained from Sigma-Aldrich and Alfa Aesar and used without further purification. Column chromatography was carried out using silica gel 40–60 μM. Reaction progress was monitored with TLC using fluorescent pre-coated silica gel plates, and detection of the components was performed using short UV light (λ = 254 nm). ^1^H-NMR spectra were run at 400 MHz, and ^13^C-NMR spectra were run at 101 MHz using a Varian Mercury 400 plus in deuterated chloroform (CDCl_3_) and DMSO-*_d6_*. Chemical shifts (*δ*) are reported in parts per million (ppm), and all coupling constants (*J*) are given in Hz. Multiplicities are abbreviated as s: singlet, d: doublet, t: triplet, dd: doublet of doublet, ddd: doublet of doublet of doublet, ddt: doublet of doublet of triplet, and m: multiplet. The mass spectrometric analysis (UHPLC-ESI-MS) was performed using the Waters ACQUITY XevoTQD system, which consisted of an ACQUITY UPLC H-Class system (Waters Corp., Milford, MA, USA) and Xevo^TM^ TQD triple-quadrupole tandem mass spectrometer with an electrospray ionization (ESI) interface (Waters Corp., Milford, MA, USA). An Acquity BEH C18 100 mm × 2.1 mm column (particle size, 1.7 μm) was used to separate analytes (Waters, Ireland). The solvent system consisted of water containing 0.1% formic acid (A) and 0.1% formic acid in acetonitrile (B). HPLC method: The flow rate was 200 μL/min. The percentage of B started at 5% and was maintained for 1 min, and then increased up to 100% during 10 min, kept at 100% for 2 min, flushed back to 5% in 3 min, and then kept at 5% for 1 min. The MS scan was carried out in the following conditions: capillary voltage of 3.5 kV, cone voltage of 20 V, radio frequency (RF) lens voltage of 2.5 V, source temperature of 150 °C, and desolvation gas temperature of 500 °C. Nitrogen was used as the desolvation and cone gas at a flow rate of 1000 and 20 L/h, respectively. System operation and data acquisition were controlled using Mass Lynx 4.1 software (Waters). The purity of the tested compounds was determined using HPLC coupled with a mass spectrometer and was higher than 95% for all compounds. Melting points were determined on a Büchi B-540 Melting Point apparatus (Flawil, Switzerland) and were uncorrected. 

#### 3.1.1. General Synthetic Procedures

##### Procedure A: General Procedure for Preparation of 2-Anilinobenzoic Acid Derivatives (**A1**–**A3**)

A mixture of 2-chlorobenzoic acid (1.9 g, 12 mmol), aniline (12 mmol), potassium carbonate (3.45 g, 25 mmol), Cu powder (0.2 g), and KI (0.1 g) in 50 mL of DMF was heated under reflux overnight. After the complete reaction, DMF was evaporated under vacuum followed by extraction using methylene chloride and water. The aqueous layer was collected, filtered under vacuum and subsequently acidified using HCl: H_2_O (1:1). A white precipitate was obtained and left to dry to obtain the 2-anilinobenzoic acid derivatives in different yields. Compounds (**A1**–**A3**) were used for the next step without further purification.

***2-Anilinobenzoic acid (A1).*** The compound was synthesized according to procedure A, using 2-chlorobenzoic acid and aniline to produce a white powder (73% yield); mp 183–185 °C [41].

***2-(4-Methoxyanilino)benzoic acid (A2).*** The compound was synthesized according to procedure A, using 2-chlorobenzoic acid and 4-methoxy aniline to produce a white powder (75% yield); mp 181–183 °C [41].

***2-(4-Methylanilino)benzoic acid (A3).*** The compound was synthesized according to procedure A, using 2-chlorobenzoic acid and 4-methyl aniline to produce a white powder (51% yield); mp 195–197 °C [41].

##### Procedure B: General Procedure for Synthesis of Acridone Derivatives (**B1**–**B3**)

The appropriate compound (**A1**–**A3**) (14 mmol) was added to 10 mL of conc. H_2_SO_4_ and heated at a temperature of 100–110 °C in an oil bath for 4 h. Afterwards, the mixture was poured slowly into an ice/water mixture to produce a yellow precipitate, which was then obtained via vacuum filtration and left to dry to obtain the acridone derivatives in different yields. No further purification was required.

***10H-Acridin-9-one (B1).*** The compound was synthesized according to procedure B, using compound A1 to produce a yellow powder (48% yield); mp 348–350 °C [42].

***2-Methoxy-10H-acridin-9-one (B2).*** The compound was synthesized according to procedure B, using compound **A2** to produce a yellow powder (51% yield); mp 285–287 °C [43].

***2-Methyl-10H-acridin-9-one (B3).*** The compound was synthesized according to procedure B, using compound A3 to produce a yellow powder (46% yield); mp 332–334 °C [21].

##### Procedure C: General Procedure for Synthesis of 9-Chloroacridine Derivatives (**C1**–**C3**)

The appropriate compound (**B1**–**B3**) (10 mmol) was added to 10 mL of POCl_3_, and the mixture was heated in an oil bath at a temperature of 100–110 °C for 3 h. After completion of the reaction (confirmed using TLC), the mixture was added dropwise into an excess ice/ammonia solution mixture. Subsequently, extraction using methylene chloride was conducted, whereby the organic phase was collected and dried over anhydrous magnesium sulfate. The methylene chloride layer was then evaporated under reduced pressure to produce a light green powder. Compounds (**C1**–**C3**) were used in the next step without further purification.

***9-Chloroacridine (C1).*** The compound was synthesized according to procedure C, using compound B1 to produce a light green powder (92% yield); mp 117–119 °C [44].

***9-Chloro-2-methoxyacridine (C2).*** The compound was synthesized according to procedure C, using compound B2 to produce a light green powder (77% yield); mp 153–155 °C [45].

***9-Chloro-2-methylacridine (C3).*** The compound was synthesized according to procedure C, using compound B3 to produce a light green powder (83% yield); mp 145–147 °C [46].

##### Procedure D: General Procedure for Synthesis of 9-Benzylacridine Analogs (**1**–**31**)

The respective benzylamine (2 mmol) was added to 15 mL of absolute alcohol and potassium carbonate (0.276 g, 2 mmol), and the mixture was stirred at room temperature for 45 min. The appropriate compound (**C1**–**C3**) (1 mmol) and KI (0.415 g, 0.25 mmol) were then added. The mixture was heated under reflux overnight at 80 °C. After the completion of the reaction, excess ethanol was evaporated under vacuum, followed by extraction performed using ethyl acetate and brine. The organic phase was collected after drying over anhydrous MgSO_4_ and concentrated under vacuum. The product was purified using silica gel column chromatography. 

***Acridin-9-yl-benzyl-amine (1).*** The compound was synthesized according to procedure D, using compound **C1** and benzylamine to produce an orange powder (67% yield); the product was purified using column chromatography (EtOAc:MeOH 100:3); mp 142–144 °C; ^1^H NMR (400 MHz, CDCl_3_): δ 8.08 (dd, *J* = 8.5, 4.0 Hz, 4H), 7.70–7.62 (m, 2H), 7.39 (dd, *J* = 6.7, 4.3 Hz, 4H), 7.33 (dd, *J* = 11.6, 3.5 Hz, 3H), and 4.97 (s, 2H); ^13^C NMR (101 MHz, CDCl_3_): δ 151.38, 148.88, 139.13, 130.06, 129.06, 127.98, 127.60, 123.33, 122.82, 116.75, and 54.96; (ESI-MS) *m*/*z* = 285 (M+H)⁺.

***Acridin-9-yl-(2-methylbenzyl)-amine (2).*** The compound was synthesized according to procedure D, using compound **C1** and 2-methylbenzylamine to produce an orange powder (10% yield); the product was purified using column chromatography (EtOAc:MeOH 100:4); mp 137–139 °C; ^1^H NMR (400 MHz, CDCl_3_): δ 8.04 (t, *J* = 8.3 Hz, 4H), 7.64 (t, *J* = 7.5 Hz, 2H), 7.48 (d, *J* = 6.8 Hz, 1H), 7.32–7.28 (m, 2H), 7.27–7.21 (m, 3H), 4.94 (s, 2H), and 2.25 (s, 3H); ^13^C NMR (101 MHz, CDCl_3_): δ 151.90, 148.68, 137.09, 136.16, 130.99, 130.29, 128.82, 128.39, 128.35, 126.74, 123.29, 123.00, 116.35, 52.99, and 19.19; (ESI-MS) *m*/*z* = 299 (M+H)⁺.

***Acridin-9-yl-(3-methylbenzyl)-amine (3).*** The compound was synthesized according to procedure D, using compound **C1** and 3-methylbenzylamine to produce a yellow powder (35% yield); the product was purified using column chromatography (EtOAc:MeOH 100:4); mp 116–118 °C; ^1^H NMR (400 MHz, CDCl_3_): δ 8.09 (t, *J* = 9.7 Hz, 4H), 7.67 (t, *J* = 7.4 Hz, 2H), 7.38–7.32 (m, 2H), 7.30–7.26 (m, 1H), 7.24–7.13 (m, 3H), 4.92 (s, 2H), and 2.36 (s, 3H); ^13^C NMR (101 MHz, CDCl_3_): δ 151.22, 149.27, 139.14, 138.80, 129.93, 129.42, 128.95, 128.72, 128.38, 124.67, 123.28, 122.78, 116.83, 55.06, and 21.42; (ESI-MS) *m*/*z* = 299 (M+H)⁺.

***Acridin-9-yl-(4-methylbenzyl)-amine (4).*** The compound was synthesized according to procedure D, using compound **C1** and 4-methylbenzylamine to produce a yellow powder (82% yield); the product was purified using column chromatography (EtOAc:MeOH 100:4); mp 153–155 °C; ^1^H NMR (400 MHz, CDCl_3_): δ 8.07 (d, *J* = 8.8 Hz, 4H), 7.66 (t, *J* = 7.5 Hz, 2H), 7.36–7.27 (m, 4H), 7.19 (d, *J* = 7.8 Hz, 2H), 4.94 (s, 2H), and 2.37 (s, 3H); ^13^C NMR (101 MHz, CDCl_3_): δ 151.39, 148.70, 137.62, 135.88, 129.94, 129.57, 128.68, 127.41, 123.06, 122.74, 116.32, 54.50, and 20.99; (ESI-MS) *m*/*z* = 299 (M+H)⁺.

***Acridin-9-yl-(2-methoxybenzyl)-amine (5).*** The compound was synthesized according to procedure D, using compound **C1** and 2-methoxybenzylamine to produce a yellow powder (59% yield); the product was purified using column chromatography (EtOAc:MeOH 100:4); mp 106–108 °C; ^1^H NMR (400 MHz, CDCl_3_): δ 8.09 (d, *J* = 8.8 Hz, 4H), 7.66 (t, *J* = 7.5 Hz, 2H), 7.38–7.31 (m, 2H), 7.26 (t, *J* = 9.1 Hz, 2H), 6.89 (t, *J* = 8.5 Hz, 2H), 4.92 (s, 2H), and 3.77 (s, 3H); ^13^C NMR (101 MHz, CDCl_3_): δ 157.36, 152.21, 148.86, 130.00, 129.36, 129.34, 129.08, 126.99, 123.21, 123.01, 120.76, 117.37, 110.50, 55.23, and 51.08; (ESI-MS) *m*/*z* = 315 (M+H)⁺.

***Acridin-9-yl-(3-methoxybenzyl)-amine (6)***. The compound was synthesized according to procedure D, using compound **C1** and 3-methoxybenzylamine to produce a yellow powder (7% yield); the product was purified using column chromatography (EtOAc:MeOH 100:4); mp 107–109 °C; ^1^H NMR (400 MHz, CDCl_3_) δ 8.09 (d, *J* = 7.5 Hz, 2H), 7.98 (d, *J* = 6.5 Hz, 2H), 7.57 (s, 2H), 7.33–7.24 (m, 3H), 6.99 (d, *J* = 7.0 Hz, 1H), 6.95 (s, 1H), 6.87 (d, *J* = 7.2 Hz, 1H), 4.99 (s, 2H), 3.76 (s, 3H); ^13^C NMR (101 MHz, CDCl_3_) δ 160.33, 152.98, 149.01, 140.38, 130.96, 130.33, 130.03, 126.88, 123.61, 123.24, 119.56, 113.53, 112.96, 55.41, 54.09; (ESI-MS) m/z = 315 (M+H)⁺.

***Acridin-9-yl-(4-methoxybenzyl)-amine (7)***. The compound was synthesized according to procedure D, using compound **C1** and 4-methoxybenzylamine to produce a yellow powder (62% yield); the product was purified using column chromatography (EtOAc:MeOH 100:4); mp 116–118 °C; ^1^H NMR (400 MHz, CDCl_3_): δ 8.08 (t, *J* = 10.2 Hz, 4H), 7.67 (t, *J* = 7.4 Hz, 2H), 7.37–7.28 (m, 4H), 6.91 (d, *J* = 8.0 Hz, 2H), 4.91 (s, 2H), and 3.82 (s, 3H); ^13^C NMR (101 MHz, CDCl_3_): δ 159.55, 151.46, 149.16, 131.36, 130.20, 129.18, 123.43, 122.97, 116.79, 116.49, 114.58, 55.48, and 54.72; (ESI-MS) *m*/*z* = 315 (M+H)⁺.

***Acridin-9-yl-(2-fluorobenzyl)-amine (8).*** The compound was synthesized according to procedure D, using compound **C1** and 2-fluorobenzylamine to produce a yellow powder (18% yield); the product was purified using column chromatography (EtOAc:MeOH 100:3); mp 131–133 °C; ^1^H NMR (400 MHz, CDCl_3_): δ 8.09 (d, *J* = 8.4 Hz, 4H), 7.68 (t, *J* = 7.2 Hz, 2H), 7.36 (dd, *J* = 17.7, 10.8 Hz, 3H), 7.29 (d, *J* = 6.6 Hz, 1H), 7.14–7.04 (m, 2H), and 4.96 (s, 2H); ^13^C NMR (101 MHz, CDCl_3_): δ 160.83 (d, *J* = 246.5 Hz), 150.95, 149.21, 129.91, 129.81 (d, *J* = 7.7 Hz), 129.75 (d, *J* = 19.9 Hz), 129.73 (d, *J* = 8.5 Hz), 124.51 (d, *J* = 3.6 Hz), 123.66, 122.64, 117.88, 115.65 (d, *J* = 21.3 Hz), and 49.05 (d, *J* = 3.5 Hz); (ESI-MS) *m*/*z* = 303 (M+H)⁺.

***Acridin-9-yl-(3-fluorobenzyl)-amine (9).*** The compound was synthesized according to procedure D, using compound **C1** and 3-fluorobenzylamine to produce a yellow powder (8% yield); the product was purified using column chromatography (EtOAc:MeOH 100:3); mp 154–156 °C; ^1^H NMR (400 MHz, CDCl_3_): δ 8.05 (d, *J* = 8.2 Hz, 4H), 7.67 (t, *J* = 7.3 Hz, 2H), 7.35 (dd, *J* = 14.2, 7.8 Hz, 3H), 7.17 (t, *J* = 7.8 Hz, 2H), 7.08–6.99 (m, 1H), and 4.95 (s, 2H); ^13^C NMR (101 MHz, CDCl_3_): δ 160.83 (d, *J* = 246.5 Hz), 150.95, 149.21, 129.91, 129.79 (d, *J* = 4.3 Hz), 129.69 (d, *J* = 8.5 Hz), 126.21 (d, *J* = 17.0 Hz), 124.51 (d, *J* = 3.6 Hz), 123.66, 122.64, 117.88, 115.65 (d, *J* = 21.3 Hz), and 49.05 (d, *J* = 3.5 Hz); (ESI-MS) *m*/*z* = 303 (M+H)⁺.

***Acridin-9-yl-(4-fluorobenzyl)-amine (10).*** The compound was synthesized according to procedure D, using compound **C1** and 4-fluorobenzylamine to produce a yellow powder yield (51% yield); the product was purified using column chromatography (EtOAc:MeOH 100:4); mp 145–157 °C; ^1^H NMR (400 MHz, CDCl_3_): δ 8.06 (dd, *J* = 12.4, 8.9 Hz, 4H), 7.67 (t, *J* = 7.4 Hz, 2H), 7.35 (t, *J* = 7.0 Hz, 4H), 7.06 (t, *J* = 8.6 Hz, 2H), and 4.92 (s, 2H); ^13^C NMR (101 MHz, CDCl_3_): δ 162.55 (d, *J* = 246.6 Hz), 151.22, 149.05, 135.17 (d, *J* = 3.3 Hz), 130.19, 129.44 (d, *J* = 8.2 Hz), 129.20, 123.61, 122.90, 117.22, 116.05 (d, *J* = 21.5 Hz), and 54.48; (ESI-MS) *m*/*z* = 303 (M+H)⁺.

***Acridin-9-yl-(2-chlorobenzyl)-amine (11).*** The compound was synthesized according to procedure D, using compound **C1** and 2-chlorobenzylamine to produce a yellow powder yield (12% yield); the product was purified using column chromatography (EtOAc:MeOH 100:1); mp 113–115 °C; ^1^H NMR (400 MHz, CDCl_3_): δ 8.07 (d, *J* = 8.9 Hz, 4H), 7.67 (t, *J* = 7.9 Hz, 2H), 7.43 (dd, *J* = 7.8, 1.0 Hz, 1H), 7.37 (dd, *J* = 15.9, 7.8 Hz, 3H), 7.28–7.23 (m, 1H), 7.22–7.17 (m, 1H), and 5.00 (s, 2H); ^13^C NMR (101 MHz, CDCl_3_): δ 151.25, 149.02, 136.67, 133.54, 130.14, 129.98, 129.82, 129.40, 127.43, 123.76, 122.94, 117.93, and 52.89; (ESI-MS) *m*/*z* = 319 (M+H)⁺.

***Acridin-9-yl-(3-chlorobenzyl)-amine (12).*** The compound was synthesized according to procedure D, using compound **C1** and 3-chlorobenzylamine to produce a yellow powder (36% yield); the product was purified using column chromatography (EtOAc:MeOH 100:1); mp 114–116 °C; ^1^H NMR (400 MHz, CDCl_3_): δ 8.04 (t, *J* = 6.3 Hz, 4H), 7.65 (t, *J* = 7.7 Hz, 2H), 7.43 (s, 1H), 7.34 (t, *J* = 7.8 Hz, 2H), 7.29 (d, *J* = 4.8 Hz, 2H), 7.25 (d, *J* = 5.8 Hz, 1H), and 4.90 (s, 2H); ^13^C NMR (101 MHz, CDCl_3_): δ 151.22, 148.79, 141.52, 135.00, 130.40, 130.22, 128.90, 128.16, 127.75, 125.72, 123.67, 122.95, 117.43, and 54.55; (ESI-MS) *m*/*z* = 319 (M+H)⁺.

***Acridin-9-yl-(4-chlorobenzyl)-amine (13).*** The compound was synthesized according to procedure D, using compound **C1** and 4-chlorobenzylamine to produce a yellow powder (65% yield); the product was purified using column chromatography (EtOAc:MeOH 100:1); mp 145–147 °C; ^1^H NMR (400 MHz, CDCl_3_): δ 8.24 (d, *J* = 8.4 Hz, 2H), 7.63–7.55 (m, 4H), 7.48 (d, *J* = 8.4 Hz, 2H), 7.40 (d, *J* = 8.4 Hz, 2H), 7.21 (t, *J* = 7.1 Hz, 2H), and 5.06 (s, 2H); ^13^C NMR (101 MHz, CDCl_3_): δ 151.10, 149.08, 137.96, 133.88, 130.17, 129.30, 129.07, 123.67, 122.88, 117.36, and 54.54; (ESI-MS) *m*/*z* = 319 (M+H)⁺.

***Acridin-9-yl-furan-2-ylmethyl-amine (14).*** The compound was synthesized according to procedure D, using compound **C1** and furfurylamine to produce a yellow powder yield (20% yield); the product was purified using column chromatography (EtOAc:MeOH 100:4); mp 114–116 °C; ^1^H NMR (400 MHz, CDCl_3_): δ 8.10 (d, *J* = 8.7 Hz, 4H), 7.68 (t, *J* = 7.7 Hz, 2H), 7.39 (t, *J* = 7.7 Hz, 3H), 6.32–6.18 (m, 2H), and 4.87 (s, 2H); ^13^C NMR (101 MHz, CDCl_3_): δ 152.23, 150.90, 148.90, 142.53, 130.02, 129.18, 123.75, 122.77, 117.93, 110.52, 107.71, and 47.83; (ESI-MS) *m*/*z* = 275 (M+H)⁺.

***Acridin-9-yl-thiophen-2-ylmethyl-amine (15).*** The compound was synthesized according to procedure D, using compound **C1** and 2-thiophenemethylamine to produce a yellow powder (4% yield); the product was purified using column chromatography (EtOAc:MeOH 100:3); mp 121–123 °C; ^1^H NMR (400 MHz, CDCl_3_): δ 8.11 (d, *J* = 8.6 Hz, 2H), 8.03 (d, *J* = 8.5 Hz, 2H), 7.63 (t, *J* = 7.4 Hz, 2H), 7.34 (t, *J* = 7.5 Hz, 2H), 7.28 (d, *J* = 5.0 Hz, 1H), 7.05 (s, 1H), 7.02–6.97 (m, 1H), and 5.14 (s, 2H); ^13^C NMR (101 MHz, CDCl_3_): δ 151.43, 147.86, 142.11, 130.55, 129.50, 127.39, 125.84, 125.61, 123.70, 123.29, 117.19, and 49.78; (ESI-MS) *m*/*z* = 291 (M+H)⁺.

***Acridin-9-yl-(2,3-dimethoxybenzyl)-amine (16).*** The compound was synthesized according to procedure D, using compound **C1** and 2, 3-dimethoxybenzylamine to produce a yellow powder (67% yield); the product was purified using column chromatography (EtOAc:MeOH 100:5); mp 175–177 °C; ^1^H NMR (400 MHz, CDCl_3_): δ 8.14–8.07 (m, 4H), 7.66 (ddd, *J* = 10.7, 6.0, 2.7 Hz, 2H), 7.35 (ddt, *J* = 10.7, 5.3, 2.6 Hz, 2H), 6.99 (ddd, *J* = 11.6, 8.0, 3.8 Hz, 1H), 6.88 (ddd, *J* = 9.3, 7.9, 1.5 Hz, 2H), 4.93 (s, 2H), 3.89 (s, 3H), and 3.88 (s, 3H); ^13^C NMR (101MHz, CDCl_3_): δ 152.93, 151.68, 149.30, 147.21, 132.68, 130.08, 129.44, 124.56, 123.51, 123.09, 121.24, 117.57, 112.63, 61.03, 55.99, and 50.75; (ESI-MS) *m*/*z* = 345 (M+H)⁺.

***Acridin-9-yl-(2,4-dimethoxybenzyl)-amine (17).*** The compound was synthesized according to procedure D, using compound C1 and 2, 4-dimethoxybenzylamine to produce a yellow powder (47% yield); the product was purified using column chromatography (EtOAc:MeOH 100:5); mp 170–172 °C; ^1^H NMR (400 MHz, CDCl_3_): δ 8.08 (d, *J* = 8.5 Hz, 2H), 7.68 (d, *J* = 8.5 Hz, 2H), 7.38 (t, *J* = 7.4 Hz, 2H), 7.25 (d, *J* = 8.1 Hz, 1H), 7.05 (t, *J* = 7.7 Hz, 2H), 6.52 (d, *J* = 2.1 Hz, 1H), 6.44 (dd, *J* = 8.4, 2.1 Hz, 1H), 5.00 (s, 2H), 3.86 (s, 3H), and 3.78 (s, 3H); ^13^C NMR (101 MHz, CDCl_3_): δ 167.50, 161.09, 157.52, 157.47, 139.82, 133.79, 128.31, 125.25, 122.96, 119.25, 116.67, 112.25, 104.59, 99.01, 55.67, 55.56, and 47.33; (ESI-MS) *m*/*z* = 345 (M+H)⁺.

***Acridin-9-yl-(3,4-dimethoxybenzyl)-amine (18).*** The compound was synthesized according to procedure D, using compound **C1** and 3, 4-dimethoxybenzylamine to produce a yellow powder (30% yield); the product was purified using column chromatography (EtOAc:MeOH 100:5); mp 128–130 °C; ^1^H NMR (400 MHz, CDCl_3_): δ 8.08 (t, *J* = 8.6 Hz, 4H), 7.67 (t, *J* = 7.5 Hz, 2H), 7.38–7.30 (m, 2H), 6.94 (dd, *J* = 8.1, 1.5 Hz, 1H), 6.85 (d, *J* = 8.2 Hz, 1H), 6.81 (d, *J* = 1.4 Hz, 1H), 4.90 (s, 2H), 3.88 (s, 3H), and 3.75 (s, 3H); ^13^C NMR (101 MHz, CDCl_3_): δ 151.38, 149.33, 148.99, 148.76, 131.78, 130.05, 129.16, 123.31, 122.87, 119.88, 116.78, 111.39, 110.74, 55.94, 55.82, and 54.94; (ESI-MS) *m*/*z* = 345 (M+H)⁺.

***Acridin-9-yl-(3-fluoro-4-methoxybenzyl)-amine (19).*** The compound was synthesized according to procedure D, using compound **C1** and 3-fluoro-4-methoxybenzylamine to produce a yellow powder (51% yield); the product was purified using column chromatography (EtOAc:MeOH 100:5); mp 145–147 °C; ^1^H NMR (400 MHz, CDCl_3_): δ 8.05 (t, *J* = 8.7 Hz, 4H), 7.69–7.63 (m, 2H), 7.37–7.31 (m, 2H), 7.13 (dd, *J* = 11.8, 2.1 Hz, 1H), 7.06 (d, *J* = 8.4 Hz, 1H), 6.95–6.88 (m, 1H), 4.86 (s, 2H), and 3.88 (s, 3H); ^13^C NMR (101 MHz, CDCl_3_): δ 152.47 (d, *J* = 247.2 Hz), 151.04, 148.81, 147.27 (d, *J* = 10.7 Hz), 132.20 (d, *J* = 5.7 Hz), 130.12, 128.98, 123.41, 123.32 (d, *J* = 3.6 Hz), 122.80, 117.07, 115.40 (d, *J* = 18.7 Hz), 113.71 (d, *J* = 2.2 Hz), 56.30, and 54.12 (d, *J* = 1.3 Hz); (ESI-MS) *m*/*z* = 333 (M+H)⁺.

***Acridin-9-yl-(2,3-difluorobenzyl)-amine (20).*** The compound was synthesized according to procedure D, using compound **C1** and 2, 3-difluorobenzylamine to produce a yellow powder (9% yield); the product was purified using column chromatography (EtOAc:MeOH 100:4); mp 114–116 °C; ^1^H NMR (400 MHz, CDCl_3_): δ 8.05 (dd, *J* = 17.5, 8.6 Hz, 4H), 7.66 (t, *J* = 7.8 Hz, 2H), 7.37 (t, *J* = 7.7 Hz, 2H), 7.12 (t, *J* = 8.2 Hz, 2H), 7.04–6.98 (m, 1H), and 4.99 (s, 2H); (ESI-MS) *m*/*z* = 321 (M+H)⁺.

***Acridin-9-yl-(2,4-difluorobenzyl)-amine (21).*** The compound was synthesized according to procedure D using compound **C1** and 2, 4-difluorobenzylamine to produce a yellow powder: yield (26%); the product was purified using column chromatography (EtOAc:MeOH 100:4); mp 127–129 °C; ^1^H NMR (400 MHz, CDCl_3_): δ 8.06 (d, *J* = 8.5 Hz, 4H), 7.67 (t, *J* = 7.5 Hz, 2H), 7.41–7.34 (m, 2H), 7.30–7.24 (m, 1H), 6.87–6.76 (m, 2H), and 4.90 (s, 2H); ^13^C NMR (101 MHz, CDCl_3_): δ 162.93 (dd, *J* = 177.2, 12.1 Hz), 160.45 (dd, *J* = 177.0, 12.0 Hz), 150.84, 148.85, 130.56 (dd, *J* = 9.7, 6.0 Hz), 129.98, 123.70, 122.75, 118.06, 111.53 (dd, *J* =21.2, 3.8 Hz), 104.16 (t, *J* = 25.4 Hz), and 48.49 (d, *J* = 3.0 Hz); (ESI-MS) *m*/*z* = 321 (M+H)⁺.

***Acridin-9-yl-(3,4-difluorobenzyl)-amine (22).*** The compound was synthesized according to procedure D, using compound **C1** and 3, 4-difluorobenzylamine to produce a yellow powder (32% yield); the product was purified using column chromatography (EtOAc:MeOH 100:4); mp 146–148 °C; ^1^H NMR (400 MHz, CDCl_3_): δ 8.03 (d, *J* = 8.2 Hz, 4H), 7.65 (t, *J* = 7.4 Hz, 2H), 7.34 (t, *J* = 7.4 Hz, 2H), 7.25 (d, *J* = 6.0 Hz, 1H), 7.14 (dd, *J* = 17.1, 8.2 Hz, 2H), and 4.88 (s, 2H); ^13^C NMR (101 MHz, CDCl_3_): δ 150.52 (dd, *J* = 249.9, 12.5 Hz), 149.78 (dd, *J* = 238.3, 10.2 Hz), 148.38, 136.50 (dd, *J* = 8.6, 2.4 Hz), 130.08, 123.52, 123.34 (dd, *J* = 6.3, 3.6 Hz), 122.84, 117.70 (dd, *J* = 17.5, 9.1 Hz), 116.44 (dd, *J* = 17.4, 8.5 Hz), and 54.00; (ESI-MS) *m*/*z* = 321 (M+H)⁺.

***Acridin-9-yl-phenethyl-amine (23).*** The compound was synthesized according to procedure D, using compound **C1** and phenylethylamine to produce a yellow solid (50% yield); the product was purified using column chromatography (EtOAc:MeOH 100:4); mp 151–153 °C; ^1^H NMR (400 MHz, CDCl_3_): δ 8.06 (d, *J* = 1.2 Hz, 1H), 8.04 (d, *J* = 1.2 Hz, 1H), 7.94 (d, *J* = 1.3 Hz, 1H), 7.92 (d, *J* = 1.3 Hz, 1H), 7.63–7.58 (m, 2H), 7.36–7.31 (m, 2H), 7.28 (dd, *J* = 3.4, 1.3 Hz, 1H), 7.27–7.22 (m, 4H), 4.04 (t, *J* = 7.0 Hz, 2H), and 3.01 (t, *J* = 7.0 Hz, 2H); ^13^C NMR (101 MHz, CDCl_3_): δ 151.35, 148.61, 137.94, 130.04, 128.89, 128.87, 128.67, 126.94, 123.02, 122.87, 116.69, 51.40, and 37.37; (ESI-MS) *m*/*z* = 298 (M+H)⁺.

***Acridin-9-yl-(4-thiophen-2-yl-benzyl)-amine (24).*** The compound was synthesized according to procedure D, using compound **C1** and 4-(2-thienyl)benzylamine to produce a yellow powder (21% yield); the product was purified using column chromatography (EtOAc:MeOH 100:3); mp 164–166 °C; ^1^H NMR (400 MHz, CDCl_3_): δ 8.08 (dd, *J* = 8.5, 3.2 Hz, 4H), 7.69–7.63 (m, 2H), 7.61 (d, *J* = 8.2 Hz, 2H), 7.39 (d, *J* = 8.1 Hz, 2H), 7.33 (dd, *J* = 10.0, 5.4 Hz, 3H), 7.29 (d, *J* = 5.1 Hz, 1H), 7.09 (dd, *J* = 5.0, 3.7 Hz, 1H), and 4.97 (s, 2H); ^13^C NMR (101 MHz, CDCl_3_): δ 151.51, 148.52, 143.69, 138.26, 134.10, 130.18, 128.66, 128.09, 128.07, 126.45, 125.04, 123.34, 123.29, 122.94, 116.68, and 54.50; (ESI-MS) *m*/*z* = 367 (M+H)⁺.

***Benzyl-(2-methoxyacridin-9-yl)-amine (25).*** The compound was synthesized according to procedure D, using compound **C2** and benzylamine to produce a yellow powder (17% yield); the product was purified using column chromatography (EtOAc:MeOH 100:2); mp 123–125 °C; ^1^H NMR (400 MHz, CDCl_3_): δ 8.12 (d, *J* = 8.7 Hz, 1H), 8.03 (dd, *J* = 8.7, 6.2 Hz, 2H), 7.69–7.59 (m, 1H), 7.42 (d, *J* = 7.2 Hz, 2H), 7.40–7.36 (m, 3H), 7.36–7.29 (m, 2H), 7.20 (d, *J* = 2.2 Hz, 1H), 4.85 (s, 2H), and 3.75 (s, 3H); ^13^C NMR (101 MHz, CDCl_3_): δ 156.00, 149.56, 147.88, 146.32, 139.68, 131.36, 129.78, 129.13, 129.08, 127.92, 127.52, 124.60, 124.20, 122.13, 118.33, 118.15, 99.53, 55.43, and 54.64; (ESI-MS) *m*/*z* = 315 (M+H)⁺.

***(2-Methoxyacridin-9-yl)-(4-methylbenzyl)-amine (26).*** The compound was synthesized according to procedure D, using compound **C2** and 4-methylbenzylamine to produce a green powder (12% yield); the product was purified using column chromatography (EtOAc:MeOH 100:2); mp 131–133 °C; ^1^H NMR (400 MHz, CDCl_3_): δ 8.11 (t, *J* = 7.7 Hz, 1H), 8.04 (dd, *J* = 9.0, 4.2 Hz, 2H), 7.67–7.61 (m, 1H), 7.41–7.34 (m, 2H), 7.30 (d, *J* = 7.9 Hz, 2H), 7.22 (d, *J* = 2.6 Hz, 1H), 7.18 (d, *J* = 7.9 Hz, 2H), 4.82 (s, 2H), 3.80 (s, 3H), and 2.36 (s, 3H); ^13^C NMR (101 MHz, CDCl_3_): δ 155.97, 149.69, 147.84, 146.25, 137.70, 136.63, 131.32, 129.75, 129.16, 127.56, 124.57, 124.12, 122.25, 118.22, 118.02, 99.62, 55.49, 54.49, and 21.28; (ESI-MS) *m*/*z* = 329 (M+H)⁺.

***(2-Methoxyacridin-9-yl)-(4-methoxybenzyl)-amine (27).*** The compound was synthesized according to procedure D, using compound **C2** and 4-methoxybenzylamine to produce a green powder (9% yield); the product was purified using column chromatography (EtOAc:MeOH 100:2); mp 168–170 °C; ^1^H NMR (400 MHz, CDCl_3_): δ 8.10 (d, *J* = 8.7 Hz, 1H), 8.03 (dd, *J* = 8.8, 5.3 Hz, 2H), 7.68–7.59 (m, 1H), 7.41–7.33 (m, 2H), 7.31 (d, *J* = 8.6 Hz, 2H), 7.23 (d, *J* = 2.6 Hz, 1H), 6.90 (t, *J* = 5.7 Hz, 2H), 4.80 (s, 2H), and 3.81 (d, *J* = 1.7 Hz, 6H); ^13^C NMR (101 MHz, CDCl_3_): δ 159.40, 155.96, 149.73, 147.70, 146.07, 131.68, 131.11, 129.45, 129.22, 128.88, 124.58, 124.09, 122.32, 118.15, 117.93, 114.45, 99.67, 55.53, 55.47, and 54.17; (ESI-MS) *m*/*z* = 345 (M+H)⁺.

***(4-Fluorobenzyl)-(2-methoxyacridin-9-yl)-amine (28).*** The compound was synthesized according to procedure D, using compound **C2** and 4-fluorobenzylamine to produce a green powder (13% yield); the product was purified using column chromatography (EtOAc:MeOH 100:2); mp 163–165 °C; ^1^H NMR (400 MHz, CDCl_3_): δ 8.11 (d, *J* = 8.7 Hz, 1H), 8.02 (dd, *J* = 16.4, 9.1 Hz, 2H), 7.64 (t, *J* = 7.4 Hz, 1H), 7.42–7.32 (m, 4H), 7.19 (d, *J* = 2.3 Hz, 1H), 7.05 (t, *J* = 8.6 Hz, 2H), 4.82 (s, 2H), and 3.80 (s, 3H); ^13^C NMR (101 MHz, CDCl_3_): δ 162.47 (d, *J* = 246.5 Hz), 156.14, 149.41, 147.67, 135.39 (d, *J* = 3.2 Hz), 131.19, 129.61, 129.27, 129.19 (d, *J* = 8.1 Hz), 124.68, 124.34, 122.10, 118.39, 118.16, 115.96 (d, *J* = 21.5 Hz), 99.47, 55.50, and 53.85; (ESI-MS) *m*/*z* =333 (M+H)⁺.

***(4-Chlorobenzyl)-(2-methoxyacridin-9-yl)-amine (29).*** The compound was synthesized according to procedure D, using compound **C2** and 4-chlorobenzylamine to produce a yellow powder (21% yield); the product was purified using column chromatography (EtOAc:MeOH 100:2); mp 178–180 °C; ^1^H NMR (400 MHz, DMSO-*_d6_*): δ 8.26 (d, *J* = 8.7 Hz, 1H), 7.87–7.80 (m, 2H), 7.61 (t, *J* = 7.5 Hz, 1H), 7.56 (s, 1H), 7.49 (d, *J* = 8.0 Hz, 2H), 7.42–7.35 (m, 3H), 7.31 (t, *J* = 7.6 Hz, 1H), 4.95 (s, 2H), and 3.75 (s, 3H); ^13^C NMR (101 MHz, DMSO-*_d6_*): δ 154.80, 150.20, 146.58, 139.35, 131.45, 129.21, 128.74, 128.37, 123.90, 122.65, 116.99, 116.37, 101.15, 55.33, and 51.62; (ESI-MS) *m*/*z* = 349 (M+H)⁺.

***Benzyl-(2-methylacridin-9-yl)-amine (30).*** The compound was synthesized according to procedure D, using compound **C3** and benzylamine to produce a yellow powder (8% yield); the product was purified using column chromatography (EtOAc:MeOH 100:2); mp 156–158 °C; ^1^H NMR (400 MHz, CDCl_3_): δ 8.11–8.00 (m, 4H), 7.51 (d, *J* = 8.8 Hz, 1H), 7.40 (d, *J* = 6.3 Hz, 4H), 7.37 (d, *J* = 6.6 Hz, 1H), 7.35–7.30 (m, 2H), 4.94 (s, 2H), and 2.50 (s, 3H); ^13^C NMR (101 MHz, CDCl_3_): δ 150.78, 148.55, 139.40, 133.40, 132.95, 129.87, 129.17, 129.00, 128.09, 127.75, 123.49, 123.02, 121.16, 117.14, 117.08, 55.04, and 22.16; (ESI-MS) *m*/*z* = 299 (M+H)⁺.

***(4-Chlorobenzyl)-(2-methyl-acridin-9-yl)-amine (31).*** The compound was synthesized according to procedure D, using compound **C3** and 4-chlorobenzylamine to produce a yellow powder (9% yield); the product was purified using column chromatography (EtOAc:MeOH 100:2); mp 126–128 °C; ^1^H NMR (400 MHz, CDCl_3_): δ 8.07 (d, *J* = 8.7 Hz, 1H), 8.01 (dd, *J* = 8.6, 3.6 Hz, 2H), 7.77 (s, 1H), 7.67–7.61 (m, 1H), 7.52 (dd, *J* = 8.8, 1.5 Hz, 1H), 7.36–7.31 (m, 5H), 4.87 (s, 2H), and 2.50 (s, 3H); ^13^C NMR (101 MHz, CDCl_3_): δ 150.14, 148.57, 137.85, 133.66, 133.43, 132.71, 129.62, 129.24, 129.10, 128.89, 123.52, 122.68, 120.81, 117.33, 117.27, 54.23, and 22.03; (ESI-MS) *m*/*z* = 333 (M+H)⁺.

### 3.2. Biology

#### 3.2.1. Cell Lines, Biomaterials, and Chemicals

The cell lines used were obtained from The American Type Culture Collection (ATCC, Manassas, VA, USA). The cell lines were stored at 37 °C in a humidified atmosphere of 5% CO_2_. McCoy’s 5a Medium Modified was used as a growth medium for HCT-116, and fetal bovine (10%) serum was used to supplement the growth medium. Annexin V-FITC/PI (propidium iodide) stain was supplied by Strong Biotech Corporation (Taipei, Taiwan). Dimethyl sulfoxide (DMSO), 3-(4,5-dimethylthiazol-2-yl)-2,5-diphenyl-tetrazolium bromide (MTT), and all other chemicals were purchased from Sigma-Aldrich (St. Louis, MO, USA).

#### 3.2.2. Cell Proliferation Activity Assay

In the MTT assay, 96-well culture plates were used. A total of 7 × 10^4^ cells per well were seeded, and test materials were added to the cells at various concentrations [47,48]. Human cancer and non-cancer cell lines were purchased commercially from the American Type Culture Collection (ATCC). After 72 h, the cytotoxicity of the compounds and paclitaxel (as a positive control group) was detected using the MTT method (thiazolyl blue tetrazolium bromide, Sigma-M2128). Absorbance values at 570 and 620 nm (OD = OD570–OD620) were measured using an ELISA reader (Anthos Labtec Instruments, Salzburg, Austria). IC_50_ values were determined for compounds that showed more than 50% growth inhibition at 10 µM, using concentrations of 1, 2.5, 5, and 10 µM. 

#### 3.2.3. Apoptosis Assay

To assess the mechanism of HCT-116 cell death induced by the compounds, an apoptosis assay was conducted with a flow cytometer using a FITC Annexin-V apoptosis detection kit (BD, Biosciences). The number of apoptotic/necrotic cells was analyzed using flow cytometry following the manufacturer’s protocol. Markers were assessed using a FACS-Caliburflow cytometer (Beckman Coulter, Taipei, Taiwan) and analyzed using WinMDI 2.8 software.

#### 3.2.4. Cell Cycle Arrest Analysis

To analyze cell cycle changes, cells permeated at 1 × 10^6^ × cells/mL were trypsinized and diluted and then fixed in 70% ethanol at 4 °C. Cell cycle arrest in HCT-116 cells following compound exposure was accomplished using propidium iodide (Sigma-Aldrich, St. Louis, MI, USA). The markers were then assessed using a FACS-Caliburflow cytometer (Beckman Coulter, Taipei, Taiwan) and analyzed using WinMDI software. Markers were assessed using a FACS-Caliburflow cytometer (Beckman Coulter, Taipei, Taiwan) and analyzed using WinMDI 2.8 software [49,50].

#### 3.2.5. Determination of Topoisomerase II Inhibition

The assay was performed following the manufacturer’s protocol, using a standard relaxation reaction mix (20 μL) containing 50 mM of Tris-HCl (pH 8.0), 10 mM of MgCl_2_, 200 mM of potassium glutamate, 10 mM of dithiothreitol, 50 μg/mL of bovine serum albumin, 1 mM of ATP, 0.3 μg of pHOT1 plasmid DNA, 8 units of human topo II (Topogen, Columbus, OH, USA), and various compounds (of 1 and 10 μM) at 37 °C for 30 min. The reaction was terminated by adding 2 μL of 10% SDS, followed by 2.5 μL of proteinase K (50 μg/mL) to digest the bound protein. The system was incubated at 37 °C for 15 min. The DNA product was finally analyzed using electrophoresis on a vertical 1.5% agarose gel at 2 volts/cm in 1 × TAE buffer and photographed using the Eagle Eye II system (Stratagene, La Jolla, CA, USA).

#### 3.2.6. Phosphodiesterase 5 Assay

The enzyme activities of phosphodiesterase 5 (PDE5) were measured using an HTRF cGMP assay kit (Cisbio, Codolet, France). PDE5A (0.5 ng/μL; BPS Bioscience, San Diego, CA, USA) were incubated with DMSO (0.1%, as a control), test compounds, or tadalafil at 37 °C for 10 min, and then cGMP (500 nM) was added and incubated for another 45 min. Afterward, the HTRF reagent (cGMP-d2 and anti-cGMP-cryptate) was added for 1 h at 25 °C, and PDE activity was assayed on an HTRF reader (Tecan Infinite F200 Pro). Tadalafil was used as a positive control, and it showed an IC_50_ of 10 nM in the assay conditions. 

## 4. Conclusions

PDE5 inhibitors have been reported in several studies in the context of dually acting compounds [49,51,52,53]. Herein, we provided first-in-class compounds with dual inhibition of PDE5 and Topo II. The scaffold was found to be active against PDE5 at low micromolar concentrations, with several substituents tolerated on the benzyl moiety. The 2-methoxy substituent of the acridine proved to be an important feature for boosting the activity on a sub-micromolar level, encouraging its presence during future synthesis. Most of the synthesized 9-benzylaminoacridines possessed significant anticancer activity, as indicated by the results obtained via NCI testing. Compounds **11**, **12**, and **28**, which inhibited PDE5 at low micromolar IC_50_ values, exhibited the most potent inhibition of Topo II. Finally, active growth inhibitors had variable effects on cell cycle progression and apoptosis in HCT-116 cells, which requires further investigation of the detailed mode of action of the present series. 

## Figures and Tables

**Table 1:**
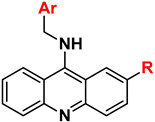
% inhibitions of the synthesized compounds against PDE5A, and IC_50_ values ^a^.

Cpd. No.	R	Ar	PDE5 % Inhibition at 10 μM	PDE5 IC_50_ (μM)
**1**	-H	phenyl	52.24 ± 5.87	9.33 ± 4.62
**2**	-H	2-tolyl	50.35 ± 3.68	9.87 ± 2.06
**3**	-H	3-tolyl	59.34 ± 6.18	6.40 ± 1.89
**4**	-H	4-tolyl	73.98 ± 12.43	4.44 ± 1.82
**5**	-H	2-methoxyphenyl	45.06 ± 4.54	>10
**6**	-H	3-methoxyphenyl	39.74 ± 3.20	>10
**7**	-H	4-methoxyphenyl	NI	>10
**8**	-H	2-fluorophenyl	57.53 ± 6.43	7.16 ± 2.75
**9**	-H	3-fluorophenyl	55.94 ± 5.78	7.49 ± 2.20
**10**	-H	4-fluorophenyl	46.97 ± 7.06	>10
**11**	-H	2-chlorophenyl	87.29 ± 3.68	4.33 ± 2.35
**12**	-H	3-chlorophenyl	62.16 ± 4.94	6.29 ± 1.18
**13**	-H	4-chlorophenyl	45.27 ± 3.99	>10
**14**	-H	2-furanyl	56.55 ± 7.38	7.43 ± 2.69
**15**	-H	2-thienyl	58.43 ± 1.47	6.43 ± 1.30
**16**	-H	2,3-dimethoxyphenyl	36.72 ± 8.14	>10
**17**	-H	2,4-dimethoxyphenyl	60.20 ± 5.89	6.77 ± 1.31
**18**	-H	3,4-dimethoxyphenyl	51.16 ± 5.26	9.77 ± 3.11
**19**	-H	3-fluoro-4-methoxyphenyl	79.24 ± 8.48	3.39 ± 1.03
**20**	-H	2,3-difluorphenyl	57.86 ± 6.04	7.25 ± 2.21
**21**	-H	2,4-difluorphenyl	31.93 ± 8.01	>10
**22**	-H	3,4-difluorphenyl	36.24 ± 9.78	>10
**23**	-H	phenylmethyl	57.31 ± 3.58	6.68 ± 1.10
**24**	-H	4-(thiophen-2-yl)phenyl	42.94 ± 5.35	>10
**25**	-OCH_3_	phenyl	77.23 ± 3.03	3.61 ± 1.00
**26**	-OCH_3_	4-tolyl	88.09 ± 4.28	0.83 ± 0.12
**27**	-OCH_3_	4-methoxyphenyl	88.77 ± 4.84	1.87 ± 1.03
**28**	-OCH_3_	4-fluorophenyl	64.25 ± 7.47	5.31 ± 1.27
**29**	-OCH_3_	4-chlorophenyl	66.07 ± 3.27	5.35 ± 1.01
**30**	-CH_3_	phenyl	68.75 ± 7.05	4.52 ± 1.74
**31**	-CH_3_	4-chlorophenyl	65.67 ± 7.48	4.78 ± 1.48

^a^ Results are presented as means ± S.D. (*n* = 4); NI, no inhibition.

**Table 2:** NCI mean % growth inhibition values.

Cpd. No.	Mean % Growth Inhibition	Cpd. No.	Mean % Growth Inhibition
1	62.74	17	60.80
2	77.65	18	81.58
3	77.21	19	76.16
4	74.79	20	40.73
5	60.77	21	56.35
6	63.07	22	64.84
7	63.25	23	60.71
8	56.47	24	96.88
9	64.19	25	80.05
10	72.34	26	79.9
11	78.36	27	87.94
12	72.98	28	92.16
13	59.43	29	93.27
14	38.41	30	64.5
16	63.87	31	66.88

**Table 3:** Cytotoxic activity of the synthesized compounds against HCT-116 and non-cancer cell lines.

Cell Line	Colorectal Adenocarcinoma		Skin Fibroblast	
	HCT-116		CCD966SK	
Cpd. No.	% Inhibition at 10 µM	IC_50_ μM ^a^	% Inhibition at 10 µM	IC_50_ μM ^a^
1	67.16 ± 2.40	2.81 ± 0.2	47.97 ± 1.96	ND
2	66.96 ± 0.14	4.01 ± 0.17	59.74 ± 0.55	4.18 ± 0.36
3	66.71 ± 2.10	4.60 ± 0.07	54.31 ± 0.98	8.51 ± 0.07
4	69.31 ± 2.09	3.59 ± 0.27	38.07 ± 5.90	ND
5	63.71 ± 0.62	6.83 ± 0.31	6.65 ± 1.18	ND
6	18.97 ± 4.43	ND	54.31 ± 0.98	8.51 ± 0.07
7	30.16 ± 9.09	ND	13.09 ± 2.52	ND
8	66.44 ± 0.80	3.05 ± 0.15	42.52 ± 2.0	ND
9	57.06 ± 1.36	7.30 ± 0.33	42.74 ± 2.09	ND
10	67.57 ± 0.29	2.75 ± 0.04	52.93 ± 1.00	9.26 ± 0.21
11	63.49 ± 1.20	7.13 ± 0.24	36.77 ± 3.49	ND
12	63.40 ± 7.54	4.84 ± 0.05	45.43 ± 0.42	ND
13	11.67 ± 4.59	ND	2.73 ± 4.11	ND
14	58.83 ± 1.72	8.41 ± 0.19	33.89 ± 0.85	ND
15	39.65 ± 4.72	ND	11.54 ± 0.04	ND
16	43.58 ± 2.65	ND	29.78 ± 2.20	ND
17	35.40 ± 3.74	ND	35.11 ± 0.42	ND
18	49.26 ± 0.88	9.74 ± 0.31	32.27 ± 0.58	ND
19	48.79 ± 1.04	ND	41.77 ± 0.99	ND
20	58.56 ± 3.21	4.96 ± 0.02	40.30 ± 3.56	ND
21	67.37 ± 0.67	3.95 ± 0.82	30.94 ± 0.86	ND
22	56.92 ± 0.61	6.99 ± 0.17	37.49 ± 7.38	ND
23	40.12 ± 0.11	ND	11.36 ± 7.38	ND
24	57.14 ± 3.10	7.00 ± 0.56	47.41 ± 2.48	ND
25	59.62 ± 2.66	7.43 ± 0.77	44.14 ± 3.80	ND
26	38.11 ± 1.35	ND	46.76 ± 3.21	ND
27	36.43 ± 7.55	ND	19.04 ± 3.94	ND
28	75.53 ± 2.52	2.51 ± 0.53	60.66 ± 2.63	4.67 ± 0.07
29	28.83 ± 2.64	ND	2.90 ± 2.51	ND
30	68.46 ± 1.55	5.78 ± 0.11	47.93 ± 1.06	ND
31	55.53 ± 1.69	7.95 ± 0.65	60.02 ± 3.07	4.96 ± 0.62
cisplatin	83.00 ± 0.38	3.62 ± 0.19	38.73 ± 5.19	ND

^a^ Data are shown as means ± SD from three independent experiments; ND, not determined.

## Data Availability

The data are contained within the article and Supplementary Material.

## Supplementary Materials

The following supporting information can be downloaded at https://www.mdpi.com/article/10.3390/molecules28020840/s1. Table S1–S3: NCI testing for compounds (**1**–**14**) and (**16**–**31**).
