# Supplementary material for: Novel 9-Benzylaminoacridine Derivatives as Dual Inhibitors of Phosphodiesterase 5 and Topoisomerase II for the Treatment of Colon Cancer
Source: Molecules. 2023 Jan 14;28(2):840. doi: 10.3390/molecules28020840 (PMC9866191; doi:10.3390/molecules28020840)
Supplement: Supplementary file 1 [file molecules-28-00840-s001.zip › molecules-2114548-SI.pdf]

Supporting Information

Novel 9-benzylaminoacridine derivatives as dual inhibitors of phosphodiesterase 5 and topoisomerase II for the treatment of colon cancer

Lina Ammar[a‡], Hung-Yu Lin [b,c‡], Shou-Ping Shih [d,e], Tsai Tsen-Ni[f], Yu-Ting Syu[g], Mohammad Abdel-Halim[a], Tsong-Long Hwang*[g,h,i,j], Ashraf H. Abadi[a]*

[a]Department of Pharmaceutical Chemistry, Faculty of Pharmacy and Biotechnology, German University in Cairo, 11835 Cairo, Egypt

[b]School of Medicine, College of Medicine, I-SHOU University, Kaohsiung 824, Taiwan.

[c]Division of Urology, Department of Surgery, E-Da Cancer & E-Da Hospital, Kaohsiung 824, Taiwan

[d]Doctoral Degree Program in Marine Biotechnology, National Sun Yat-Sen University (NSYSU), 70 Lien-Hai Road, Kaohsiung 80424, Taiwan; m6430005@hotmail.com (S.-P.S.)

[e]Doctoral Degree Program in Marine Biotechnology, Academia Sinica, 128 Academia Road, Section 2, Nankang, Taipei 11529, Taiwan

[f]Graduate Institute of Marine Biology, National Dong Hwa University, Pingtung 944, Taiwan

[g]Graduate Institute of Natural Products, College of Medicine, Chang Gung University, Taoyuan 333, Taiwan

[h]Research Center for Chinese Herbal Medicine, Graduate Institute of Health Industry Technology, College of Human Ecology, Chang Gung University of Science and Technology, Taoyuan 333, Taiwan

[i]Department of Anesthesiology, Chang Gung Memorial Hospital, Taoyuan 333, Taiwan

[j]Department of Chemical Engineering, Ming Chi University of Technology, New Taipei City 243, Taiwan

‡These authors contributed equally
*Corresponding author

***Mohammad Abdel-Halim (mohammad.abdel-halim@guc.edu.eg) and Tsong-Long Hwang (htl@mail.cgu.edu.tw)**

Content: Tables S1–S3 showing growth inhibition % for each cell line in all NCI panels by the synthesized compounds

Table S1: Growth inhibition % for each cell line in all NCI panels for compounds (1-10)

Panel	Cell line	Growth inhibition%									
		Compound #									
		**1**	**2**	**3**	**4**	**5**	**6**	**7**	**8**	**9**	**10**
**Leukemia**	CCRF-CEM	61.02	82.41	85.17	79.06	62.52	69.83	60.96	54	53.69	73.98
	HL-60(TB)	56.22	84.68	87.5	74.5	55.2	59.12	57.02	43.08	53.04	78.75
	K-562	87.68	94.96	98.12	97.01	89.94	93.43	93.03	85.83	84.27	95.21
	MOLT-4	84.5	91.15	97.68	95.71	79.29	93.59	90.62	82.73	85.14	91.32
	RPMI-8226	92.35	108.88	120.39	111	91.88	83.34	84.22	87.72	89.92	100.44
	SR	88.26	97.3	102.88	100.81	90.1	91.64	93.04	81.96	80.93	93.41
**Non-small cell lung**	A549/ATCC	71.53	80.27	77.37	73.95	65.76	69.84	71.13	65.91	70.75	76.27
	EKVX	51.89	61.56	55.55	62.13	34.33	48.18	50.3	38.3	68	64.44
	HOP-62	53.99	71.92	59.59	56.35	55.48	53.55	46.1	47.53	52.06	56.97
	HOP-92	74.14	101.03	92.95	96.76	81.77	74.54	75.45	77.27	104.06	82.84
	NCI-H226	48.3	68.54	59.43	58.51	60.14	51.93	48.61	43.56	49.38	48.25
	NCI-H23	58.82	76.62	70.32	70.7	69.08	62.68	61.2	55.87	66.14	68.35
	NCI-H322M	53.7	71.05	59.66	60.25	51.4	55.98	52.86	49.67	59.83	61.51
	NCI-H460	89.68	93.06	91.29	91.25	89.77	89.98	90.31	86.51	87.33	91.15
	NCI-H522	43.24	50.04	47.87	50.95	37.64	38.3	46.97	34.17	62.22	47.6
**Colon Cancer**	COLO 205	92.71	118.51	116.17	99.72	87.23	90.37	88.41	84.94	82.68	92.47
	HCC-2998	61.76	83.76	96.42	102.62	61.15	60.67	61.44	52	70.07	81.68
	HCT-116	91.6	92.1	90.19	88.02	87.35	90.11	90.47	86.11	86.57	87.45
	HCT-15	75.44	85.39	84.09	84.36	73.97	75.63	77.99	70.31	77.14	82.42
	HT29	80.98	91.13	94.85	89.69	82.26	81.32	80.69	76.74	85.66	83.94
	KM12	69.54	83.11	74.41	76.55	67.12	64.1	66.82	57.77	51.43	71.68
	SW-620	75.51	86.22	82.77	79.76	77.89	74.49	74.4	71.53	78.03	77.2
**CNS Cancer**	SF-268	37.37	57.13	48.03	48.06	34.93	28.76	36.65	20.11	37.85	46.75
	SF-295	62.36	70.46	74.67	68.71	62.62	64.02	60.88	48.25	74.83	72.43
	SF-539	76.04	96.7	88.27	81.9	78.78	80.62	72.46	68.86	73.14	86.87
	SNB-19	60.6	72.94	70.28	70.35	64.17	64.01	65.97	56.1	64.06	67.59
	SNB-75	35.65	54.66	63.05	58.33	36.54	43.13	38.16	28.33	36.96	57.93
	U251	61.54	78.73	76.33	73.96	61.41	61.28	63.55	53.12	64.82	74.06
**Melanoma**	LOX IMVI	80.22	90.88	93.75	88.24	81.06	80.25	79.14	73.92	87.66	90.57
	MALME-3M	62.61	84.78	85.9	83.78	54.1	55.97	59.97	55.32	49.51	74.31
	M14	67.71	86.85	77.16	72.02	59.15	64.79	62.93	46.03	61.22	65.65
	MDA-MB-435	57.21	76.08	70.94	68.84	57.41	56.97	59.8	43.75	51.14	65.15
	SK-MEL-2	39.23	59.85	61.98	57.33	26.47	32.1	39.85	21.83	18.58	56.21
	SK-MEL-28	14.46	41.69	41.16	40.52	20.04	18.8	20.39	15.95	21.53	31.83
	SK-MEL-5	36.88	66.06	80.23	69.88	40.76	42.4	37.76	39.33	39.54	57.46
	UACC-257	47.34	67.28	63.76	60.61	35.91	45.02	47.74	41.19	41.86	64.04
	UACC-62	39.05	53.3	57.25	56.61	39.47	36.54	44.24	38.44	49.66	49.81
**Ovarian Cancer**	IGROV1	70.18	85.21	80.51	80.58	73.67	71.74	72.8	63.38	74.37	78.08
	OVCAR-3	48.26	68.68	69.16	65.85	45.68	45.22	51.93	35.9	32.98	61.31

	OVCAR-4	56.78	67.14	72.07	75.53	57.83	56.39	59.67	60.04	69.54	66.82
	OVCAR-5	54.34	63.29	64.2	61.94	50.22	54.12	53.59	48.77	56.19	61.55
	OVCAR-8	73.93	79.02	79.73	78.24	68.08	68.13	72.29	68.88	64.02	77.74
	NCI/ADR-RES	62.35	75.15	74.31	73.88	65.16	62.52	61.23	62.03	69.92	70.33
	SK-OV-3	53.29	59.25	54.69	53.07	38.62	45.34	46.18	49.08	48.9	60.05
**Renal Cancer**	786-0	65.69	75.65	75.79	74.62	60.69	62.68	67.62	60.04	63.55	72.4
	A498	83.43	102.25	102.22	92.36	86.68	96.35	89.95	89.14	112.57	101.77
	ACHN	81.93	88.39	84.23	85.95	77.29	80.85	81.24	76.86	82.86	87.52
	CAKI-1	66.92	74.92	82.34	81.36	54.33	64.26	70.68	64.21	73.03	74.49
	RXF 393	58.1	92.22	101.71	89.71	69.4	86.18	72.43	51.21	67.22	99.75
	SN12C	62.53	75.67	73.87	75.06	65.26	62.06	67.82	60.38	67.21	74.3
	TK-10	51.31	61.35	61.63	64.23	44.52	48.32	50.52	41.75	35.87	69.84
	UO-31	76.18	84.51	85.26	88	66.32	72.96	77.93	72.35	76.04	86.51
**Prostate Cancer**	PC-3	62.76	74.43	75.06	75.72	56.77	61.12	66.02	52.85	50.98	73.11
	DU-145	51.36	69.41	66.1	66.5	49.54	46.68	51.97	38.1	58.28	65.11
**Breast Cancer**	MCF7	88.02	93.07	93.77	90.96	83.15	85.42	87.41	83.82	92.1	92.91
	MDA-MB-231/ATCC	42.1	60.09	61.31	56.78	42.37	50.15	52.57	46.04	69.44	61.72
	HS 578T	43.89	64.32	69.66	61.48	37.95	44.39	41.98	30.93	36.15	60.48
	BT-549	27.22	44.08	32.74	28.7	23.04	31.34	19.91	15.06	22.91	19.26
	T-47D	62.25	75.45	74.68	71.94	53.84	54.07	55.1	55.17	57.34	68.91
	MDA-MB-468	80.65	94.16	100.39	92.82	71.68	86.43	72.89	78.24	99.39	88.51
**Mean growth inhibition %**		**62.74**	**77.65**	**77.21**	**74.79**	**60.77**	**63.07**	**63.25**	**56.47**	**64.19**	**72.34**

Table S2: Growth inhibition % for each cell line in all NCI panels for compounds (11-14) and (16-21)

Panel	Cell line	Growth inhibition%									
		Compound #									
		11	12	13	14	16	17	18	19	20	21
Leukemia	CCRF-CEM	89.33	81.93	69.8	42.85	60.52	44.65	69.52	70.36	37.56	46.14
	HL-60(TB)	86.37	90.98	55.33	6.96	-	63.12	87.34	87.07	23.98	41.67
	K-562	94.67	94.97	95.66	55.31	84.53	86.84	91.19	95.19	56.64	77.54
	MOLT-4	94.86	98.87	95.37	72.78	82.15	81.54	89.43	91.08	71.63	76.16
	RPMI-8226	126.92	123.54	91.98	43.4	83.03	65.14	99.38	105.78	47.06	71.69
	SR	99.51	103.9	97.68	65.98	82.52	81.94	91.43	95.15	61.82	74.78
Non-small cell lung	A549/ATCC	80.81	72.12	60.69	38.14	69.69	64.81	83.72	77.22	43.31	59.91
	EKVX	74.13	72.79	37.38	37.95	63.72	48.9	84.76	73.88	37.13	54.54
	HOP-62	59.12	54.46	47	30.37	48.84	52.83	65.54	55.54	27.3	42.76
	HOP-92			74.46		102.4	113.58	109.67		97.33	102.61
	NCI-H226	61.12	51.97	24.54	37.08	48.41	53.69	72.05	57.01	27.14	45.25
	NCI-H23	78.43	68.04	47.05	36.41	67.58	65.11	84.9	73	39.39	57.2
	NCI-H322M	72.97	68.67	45.93	34.98	58.44	60	70.23	68.35	36.57	50.84
	NCI-H460	91.17	85.77	83.63	43.48	88.38	83.35	93.83	93.66	51.99	80.01
	NCI-H522	71.51	58.32	33.49	52.67	59.93	59.37	81.8	63.76	46.15	57.7
Colon Cancer	COLO 205	99.48	99.46	91.42	64.76	78.55	75.38	94.7	91.3	59.06	73.19
	HCC-2998	117.22	153.58	110.17	31.09	61.67	64.37	69.93	94.17	41.57	58.7
	HCT-116	91.9	91.31	84.3	75.95	88.2	87.24	87.85	87.66	82.48	85.8
	HCT-15	86.41	85.97	76.74	55.78	80.23	74.04	89.75	89	60.51	71.34
	HT29	94.46	91.38	84.96	64.94	85.58	86.21	95.56	87.85	70.92	78.15
	KM12	77.98	59.16	64.38	14.49	47.63	45.7	68.4	67.22	13.05	39.96
	SW-620	90.39	88.02	69.02	57.73	74.64	79.35	83.95	83.44	58.56	70.17
CNS Cancer	SF-268	53.4	39.4	32.14	14.26	38.27	34.33	60.41	50.92	16.61	31.38
	SF-295	78.32	79.16	40.39	46.15	66.97	63.73	81.9	79.86	46.25	59.58
	SF-539	93.85	90.72	69.65	34.08	81.09	85.44	84.65	81.83	44.55	67.6
	SNB-19	74.22	73.64	56.88	37.24	64.49	66.48	78.2	72.01	41.5	58.51
	SNB-75	56.9	31.3	31.61	18.47	32.01	35.48	75.16	57.5	21.16	18.94
	U251	76.78	77.28	53.8	34.88	65.53	58.16	76.8	73.11	34.45	55.86
Melanoma	LOX IMVI	95.17	98.25	77.04	62.89	87	82.28	103.83	98.75	60.78	78.49
	MALME-3M	80.98	55.01	60.42	9.63	49.42	32.99	92.09	76.41	5.87	30.51
	M14	81.93	64.55	46.68	20.28	65.54	56.19	82.3	80.13	29.39	44.74
	MDA-MB-435	72.8	53.42	39.34	12.42	53.69	61.28	70.63	92.4	12.68	33.51
	SK-MEL-2	44.37	22.73	27.55	-1.22	20.84	5.98	61.39	54.87	-6.76	0.35
	SK-MEL-28	49.54	33.78	19.67	2.28	33.89	30.69	64.84	53.31	3.12	9.61
	SK-MEL-5	58.19	33.81	38.75	15.72	44.19	29.65	82.52	63.5	16.97	30.58
	UACC-257	55.69	54.62	23.86	18.28	45.25	25.44	77.87	62.18	7.14	31.1
	UACC-62	59.04	47.45	29.27	33.91	48.68	45.51	73.19	57.55	24.28	40.91
Ovarian Cancer	IGROV1	82.24	83.51	68.35	38.15	76.82	77.55	89.07	84.24	47.3	64.33
	OVCAR-3	46.4	42.34	48.92	3.11	34.94	30.76	56.34	47.83	13.87	29.78

	OVCAR-4	86.93	81.19	66.93	43.11	63.38	60.34	78.57	73.85	50.11	108.19
	OVCAR-5	67.42	67.64	49.53	23.62	57.02	49.7	72.54	63.07	26.75	48.33
	OVCAR-8	76.25	68.92	68.12	52.51	60.76	62.18	76.15	70.61	49.18	59.76
	NCI/ADR-RES	76.21	73.67	63.35	55.24	66.78	64.36	80.67	79.19	51.46	63.77
	SK-OV-3	45.13	36.09	40.04	29.12	33.98	36.69	58.85	51.95	24.15	40.22
**Renal Cancer**	786-0	75.49	74.83	64.73	34.27	61.19	60.92	74.59	74.01	34.64	52.41
	A498	105.85	103.08	79.22	79.89	104.13	111.48	112.1	106.26	100.79	104.38
	ACHN	87.8	84.31	77.25	73.85	78.92	74.22	95.89	89.12	69.42	76.55
	CAKI-1	74.83	71.77	58.57	59	66.32	63.82	89.47	78.66	54.29	64.71
	RXF 393	84.98	80.98	70.33	38.52	70.07	66.62	96.42	88.92	40.5	65.54
	SN12C	72.15	76.38	60.86	46.48	69.06	68.99	77.54	74.11	50.98	62.77
	TK-10	57.5	47.92	50.3	11.6	40.5	45.64	67.71	63.87	10.78	39
	UO-31	82.51	83.95	72.96	60.53	74.78	71.96	87.91	86.52	59.95	69.65
**Prostate Cancer**	PC-3	82.91	76.5	61.34	33	62.38	52.9	78.02	69.55	42.93	31.13
	DU-145	68.18	68.35	45.2	23.96	58.25	50.62	75.31	72.82	23.32	49.1
**Breast Cancer**	MCF7	100.21	99.09	83.98	78.72	89.91	85.54	99.33	97.66	70.25	89.82
	MDA-MB-231/ATCC	71.03	70.56	45.22	39.49	58.06	56.38	80.62	70.85	41.84	55.62
	HS 578T	50.03	46.33	46.2	16	35.72	31.08	62.8	54.47	13.03	24.57
	BT-549	61.88	40.86	29.14	2.59	40.28	33.26	57.88	50.52	9.44	17.2
	T-47D	69.55	59.12	56.61	30.71	52.45	47.12	74.17	64.96	31.69	52.64
	MDA-MB-468	127.94	118.42	70.41	70.65	98.92	95.17	122.71	118.67	78.01	104.03
**Mean growth inhibition %**		**78.36**	**72.98**	**59.43**	**38.41**	**63.87**	**60.80**	**81.58**	**76.16**	**40.73**	**56.35**

Table S3: Growth inhibition % for each cell line in all NCI panels for compounds (22-31)

Panel	Cell line	Growth inhibition%									
		Compound #									
		**22**	**23**	**24**	**25**	**26**	**27**	**28**	**29**	**30**	**31**
**Leukemia**	CCRF-CEM	58.48	46.72	97.43	66.58	92.91	80.61	81.65	91.57	65.07	81.83
	HL-60(TB)	65.53	58.71	156.73	91.82	99.96	120.72	134.62	164.46	59.42	
	K-562	87.66	74.99	139.89	94.25	97.29	99.73	98.05	130.19	89.05	97.37
	MOLT-4		75.37	98.04	91.99	99.7	100.15	99.46	108.25		99.9
	RPMI-8226	73.13	80.45	144.36	130.87	146.22	147.7	142.96	144.85	93.18	128.46
	SR	83.03	79.12	143.66	95.73	112.68	115.55	101.12	129.59	95.9	109.22
**Non-small cell lung**	A549/ATCC	70.93	67.28	73.34	81.78	80.67	85.36	80.33	76.36	59.83	59.16
	EKVX	64.55	63.73	41	73.14	64.8	75.83	77.57	66.22	50.36	44.58
	HOP-62	48.69	44.91	45.4	66.39	60.95	65.94	71.77	57.63	56.45	34.75
	HOP-92	108.56	101.55	102.1	113.35			111.34		106.69	104.63
	NCI-H226	52.38	43.72	30.98	65.62	57.91	66.22	70.02	51.18	50.19	27.69
	NCI-H23	64.83	61.38	61.82	78.48	71.73	78.63	85.98	71.7	64.18	54.02
	NCI-H322M	61.01	53.41	49.4	70.51	69.66	76.49	77.39	75.36	59.15	44.13
	NCI-H460	85.4	84.67	87.07	93.69	89.97	93.52	94.53	87.45	82.91	70.51
	NCI-H522	62.97	60.2	71.14	65.41	73.57	73.79	81.77	65.29	62.99	45.8
**Colon Cancer**	COLO 205	83.91	78.82	94.87	119.12	140.59	151.4	154.4	161.91	91.94	153.26
	HCC-2998	97.85	58.56	187.24	135.51	157.9	157.58	177.63	187.56	121.4	179.03
	HCT-116	91.82	83.46	182.62	85.23	91.42	87.29	91.11	93.19	90.6	85.34
	HCT-15	81.95	76.7	166.25	86.94	88.62	94.66	95.89	94.96	81.97	85.51
	HT29	86.57	77.93	146.81	91.51	98.77	102.9	99.79	102.29	88.37	94.65
	KM12	46.73	42.56	73.73	63.46	62.7	72.36	71.3	59.91	43.67	22.88
	SW-620	76.26	71.72	172.15	80.48	81.48	84.23	85.32	87.24	75.74	67.95
**CNS Cancer**	SF-268	42.82	33.45	37.13	55.76	51.58	59.75	65.37	46.47	31.03	20.85
	SF-295	70.25	65.1	84.21	80.32	76.42	80.61	85.56	74.24	71.95	56.32
	SF-539	77.92	71.94	87.49	88.16	89.28	91.54	96.15	105.77	76.79	59.77
	SNB-19	67.12	60.53	53.81	75.81	70.77	77.29	82.34	73.57	68.19	49.73
	SNB-75	33.84	32.44	63.87	54.47	53.38	72.57	76.18	42.36	34.96	33.54
	U251	69.71	58.92	172.68	77.12	84.69	83.94	77.89	97.45	63.04	74.91
**Melanoma**	LOX IMVI	87.5	82.36	190.72	131.05	106.01	175.09	166.72	186.28	90.92	157.12
	MALME-3M	49.3	42.9	181.99	77.47	77.95	92.94	105.2	181.63	44.81	114.02
	M14	56.93	57.29	174.42	73.97	71.27	80.53	84.68	186.13	54.43	117.12
	MDA-MB-435	50.79	44.74	183.45	66.2	57.81	81	76.12	75.92	41.79	29.7
	SK-MEL-2	10.58	13.69	19.21	52.12	30.04	59.07	64.97	28.71	0.04	-1.95
	SK-MEL-28	17.59	32.33	185.98	50.33	33.56	58.66	64.74	92.93	21.99	24.46
	SK-MEL-5	33.77	35.32	190.73	81.22	65.57	88.93	106.56	88.34	41.18	46.02
	UACC-257	34.94	44.77	137.4	72.93	107.97	93.42	111.1	152.17	43.36	102.88
	UACC-62	39.97	46.84	35.54	54.09	44.27	57.4	60.88	35.25	43.63	25.99
**Ovarian Cancer**	IGROV1	77.21	71.98	89.99	83.59	82.79	88.8	91.85	83.69	84.2	72.91
	OVCAR-3	31.14	28.98	90.5	58.96	53.1	59.61	68.75	48.97	33.96	29.09

	OVCAR-4	64.61	64.99	84.71	59.16	81.51	81.59	86.37	75.47	67.17	62.46
	OVCAR-5	55.36	46.6	32.87	71	66.03	69.31	81.95	73.49	59.56	49.26
	OVCAR-8	67.88	61.69	44.38	71.85	71.33	74.61	80.55	70.34	66.48	47.63
	NCI/ADR-RES	72.36	66.55	88.78	81.66	76.18	83.78	84.88	77.09	70.17	57.99
	SK-OV-3	44.34	37.41	35.05	55.48	47.3	52.85	62.14	35.96	35.66	24.77
**Renal Cancer**	786-0	68.55	61.93	69.95	77.71	84.24	84.98	93.16	176.89	65.66	65.34
	A498	90.22	105.19	102.45	114.33	101.58	105.96	108.16	91.42	88.59	87
	ACHN	80.74	78.38	41.38	87.24	83.73	89.76	94.35	82.92	76.81	66.73
	CAKI-1	74.69	69.7	64.53	74.25	73.77	80.06	92.84	78.62	64.46	54.3
	RXF 393	92.63	64.74	114.29	94.14	93.83	102.57	118.88	124.23	98.68	70.96
	SN12C	70.26	66.89	36.83	77.55	76.4	79.18	83	77.88	74.52	60.45
	TK-10	48.94	46.75	34.88	61.28	53.01	66.36	62.22	47.15	24.74	17.61
	UO-31	84.45	75.79	72.05	86.23	86.65	93.76	108.18	98.14	81.73	75.48
**Prostate Cancer**	PC-3	64.29	64.33	75.99	71.35	88.59	94.47	82.91	80.54	70.33	60.86
	DU-145	60.52	62.05	38.28	77.51	78.41	79.96	83.54	78.5	64.16	48.59
**Breast Cancer**	MCF7	87.06	87.79	162.97	95.71	96.57	129.83	127.73	156.36	88.79	120.77
	MDA-MB-231/ATCC	65.17	56.67	61.66	72.24	66.36	76.36	83.26	75.65	64.17	62.28
	HS 578T	35.22	26.72	39.41	74.79	57.29	74.09	73.89	64.05	30.22	22.24
	BT-549	31.83	26.44	66.33	49.72	48.42	56.98	65.05	49.76	31.04	24.44
	T-47D	52.1	51.09	28.07	64.69	69.4	65.81	63.52	50.73	36.39	42.07
	MDA-MB-468	110.74	111.38	172.55	109.89	118.61	114.66	123.88	130.64	110.99	121.69
**Mean growth inhibition %**		**64.84**	**60.71**	**96.88**	**80.05**	**79.9**	**87.94**	**92.16**	**93.27**	**64.5**	**66.88**
